# A First Quantification of Plant Endemism in the Manica Highlands (Zimbabwe–Mozambique) and the Significance of Open Habitats

**DOI:** 10.1002/ece3.73025

**Published:** 2026-04-05

**Authors:** Jonathan Timberlake, Vincent Ralph Clark

**Affiliations:** ^1^ Biodiversity Foundation for Africa East Sussex UK; ^2^ Afromontane Research Unit & Department of Geography University of the Free State: Qwaqwa Campus Phuthaditjhaba South Africa

**Keywords:** Afromontane, Chimanimani, grasslands, mount Gorongosa, Nyanga, phytogeography

## Abstract

The Manica Highlands is a tropical transboundary mountain system shared by Zimbabwe and Mozambique, covering 7400 km^2^. Although extensively botanized for over 100 years, with many known endemic plant species, these have never been comprehensively enumerated or their patterns of distribution determined. Based primarily on herbarium specimens, we present here the first quantitative assessment of plant endemics, totaling 216 taxa (including subspecies and described varieties) and representing over 9% of the estimated total flora. The endemics are not evenly distributed across the Manica Highlands—77 taxa (36%) are confined to the Chimanimani Mountains (only 9% of the total area) 22 (10%) are confined to the Nyanga–Rukotso–Serra Choa area, while 67 (31%) are distributed across the whole Manica Highlands, including 27 on Mount Gorongosa. These data necessitate spatial changes to the original description of the Chimanimani–Nyanga Centre of Floristic Endemism and its renaming to the Manica Highlands Centre. Regarding ecological preferences, 173 (80%) are confined to open habitats (notably montane grassland) despite these habitats comprising only 22% of the area. By comparison, only 36 (17%) are found in moist forest or on forest margins. Some 24 (11%) are predominantly found above 2000 m elevation. Although it is evident that the unique quartzite outcrops and soils associated with the Chimanimani Mountains are a strong local driver of endemism, there is insufficient evidence to determine other edaphic drivers at present, but we suggest that the high levels of endemism seen may be driven by the low‐nutrient and stressed nature of many of the open habitats. These findings show the significance and conservation importance of open montane habitats, which should help focus future conservation efforts across the region. Future structured fieldwork to explore the potential drivers of endemism would be very valuable, given that ecological data are very limited.

## Graphical abstract

We present here the first quantitative assessment of plant endemics from the Manica Highlands (Zimbabwe‐Mozambique), totalling 216 taxa, representing over 9% of the estimated total flora. A major finding is that 173 (80%) endemic taxa are principally or entirely confined to open montane habitats such as grassland, bare rock, crags and scrub or dwarf shrubland, despite these habitats comprising only 20%–25% of the total study area. Here we describe and enumerate the Manica Highlands Centre of Floristic Endemism as a more robust concept.

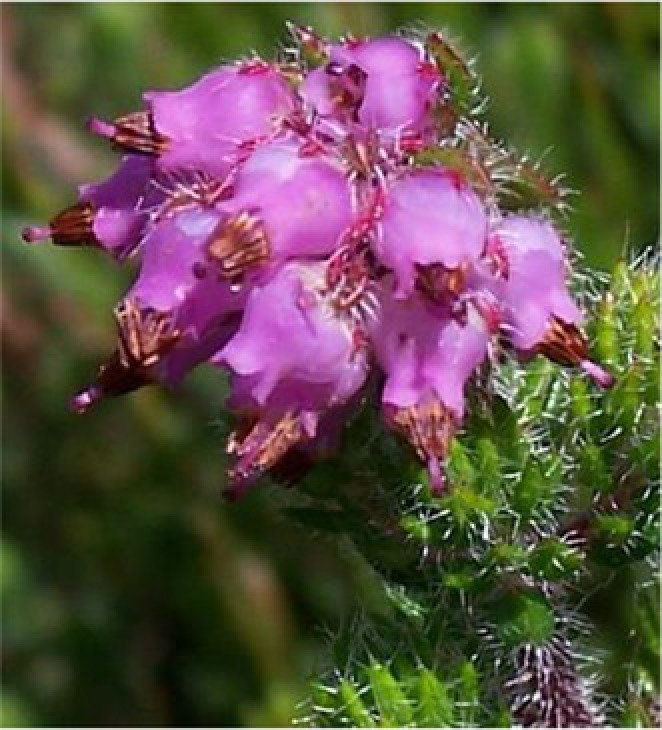

## 1 Introduction

Africa's mountains are some of the most remarkable globally due to their archipelago‐like, fragmented nature and—despite being continental in occurrence—their island‐like patterns of endemism (e.g., White 1981, 1983), which is in contrast to the extensive linear systems that dominate other continents (Hořák et al. 2023; Clark and Martin 2024). However, in comparison to better studied continents such as Europe and North America, biodiversity across African mountain systems is not well documented (e.g., Carbutt 2019; Clark et al. 2022; Hořák et al. 2023), hampering efforts to understand their ecology, focus conservation efforts, and inform sustainable development (Urbach et al. 2023).

Such knowledge is especially significant for endemic species—species confined globally to a defined area—which are of particular concern as they are national responsibilities under the Convention on Biological Diversity (Archer et al. 2018; Wilson and Primack 2019), although there is an issue here of how to share national responsibilities when species occur in transboundary ecological or geographical entities.

The Manica Highlands, a mountainous transborder area between the Zambezi and Limpopo Rivers in south‐eastern Africa and an important water source area, is not high on national conservation and policy agendas (Clark et al. 2019), which are often still focused on lowland savanna systems with large iconic animals (Mzumara et al. 2024). This is despite this series of upland massifs being of major significance in the provision of reliable water supplies for a number of surrounding towns and cities in both Mozambique and Zimbabwe (Clark et al. 2019).

Knowledge on biodiversity, particularly vegetation and plant distribution, is important when trying to select or prioritize conservation initiatives, whether these be physical construction, identification of critical habitats, control of alien invasive species or ecotourism. To help address this situation, this paper seeks to provide the first comprehensive list of vascular plant taxa endemic to the Manica Highlands. In addition, we attempt to determine the spatial patterns of endemism between its different components and consider ecological patterns, particularly as regards habitat preference, lifeform and elevation range. Building on the regional analytical approach by van Wyk and Smith (2001), which described the Chimanimani–Nyanga Centre of Floristic Endemism, we now provide a first quantitative reassessment of the endemics and a resulting spatial modification of this Centre.

### 1.1 The Manica Highlands

**FIGURE 1:**
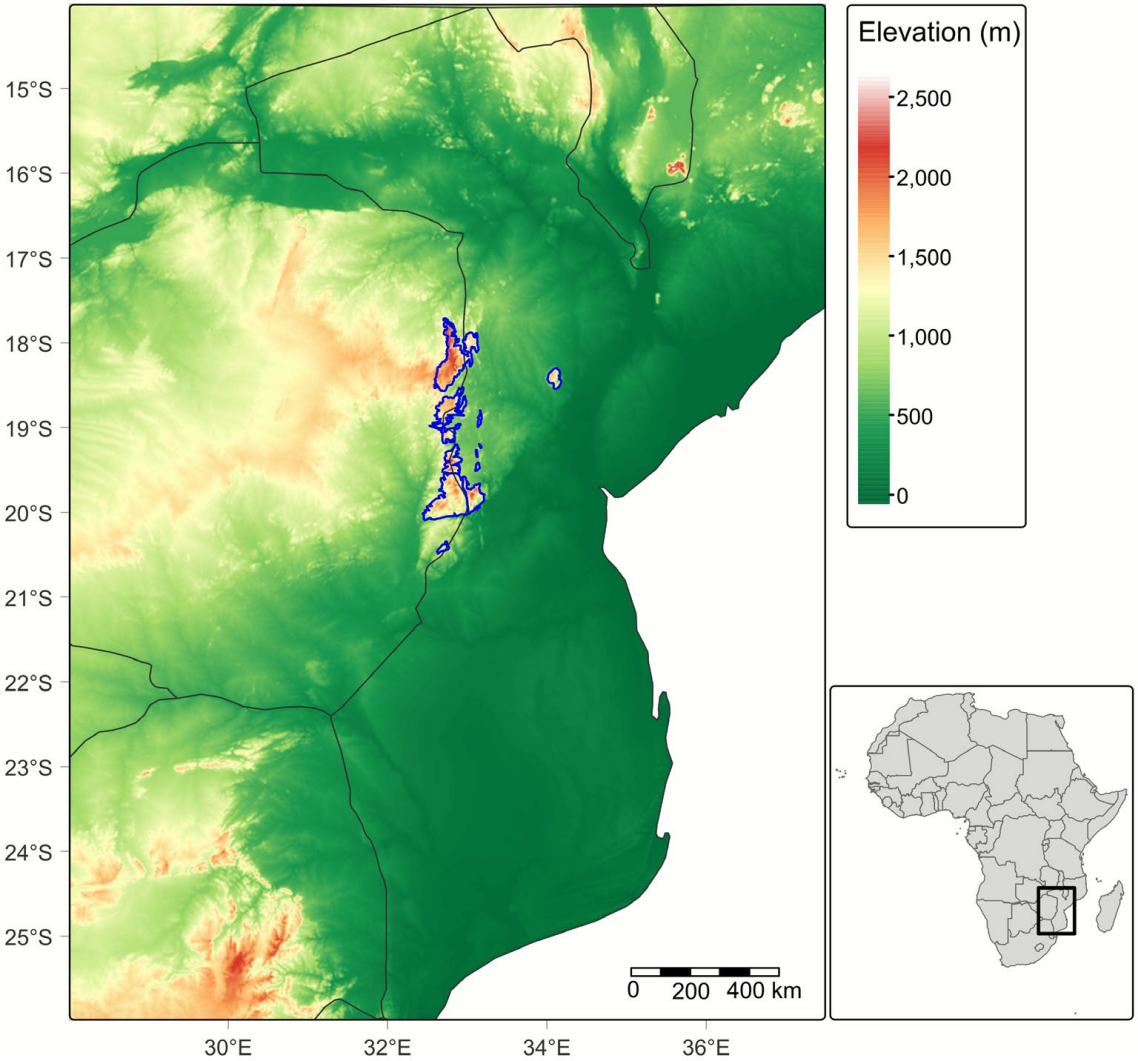
Location and extent of the Manica Highlands (Zimbabwe–Mozambique) in the context of regional elevation. The area outlined in blue represents the known distributions of montane endemic plant taxa across these mountains and is defined as a spatial representation of the Manica Highlands Centre of Floristic Endemism. Credit: Tom Timberlake.

**FIGURE 2:**
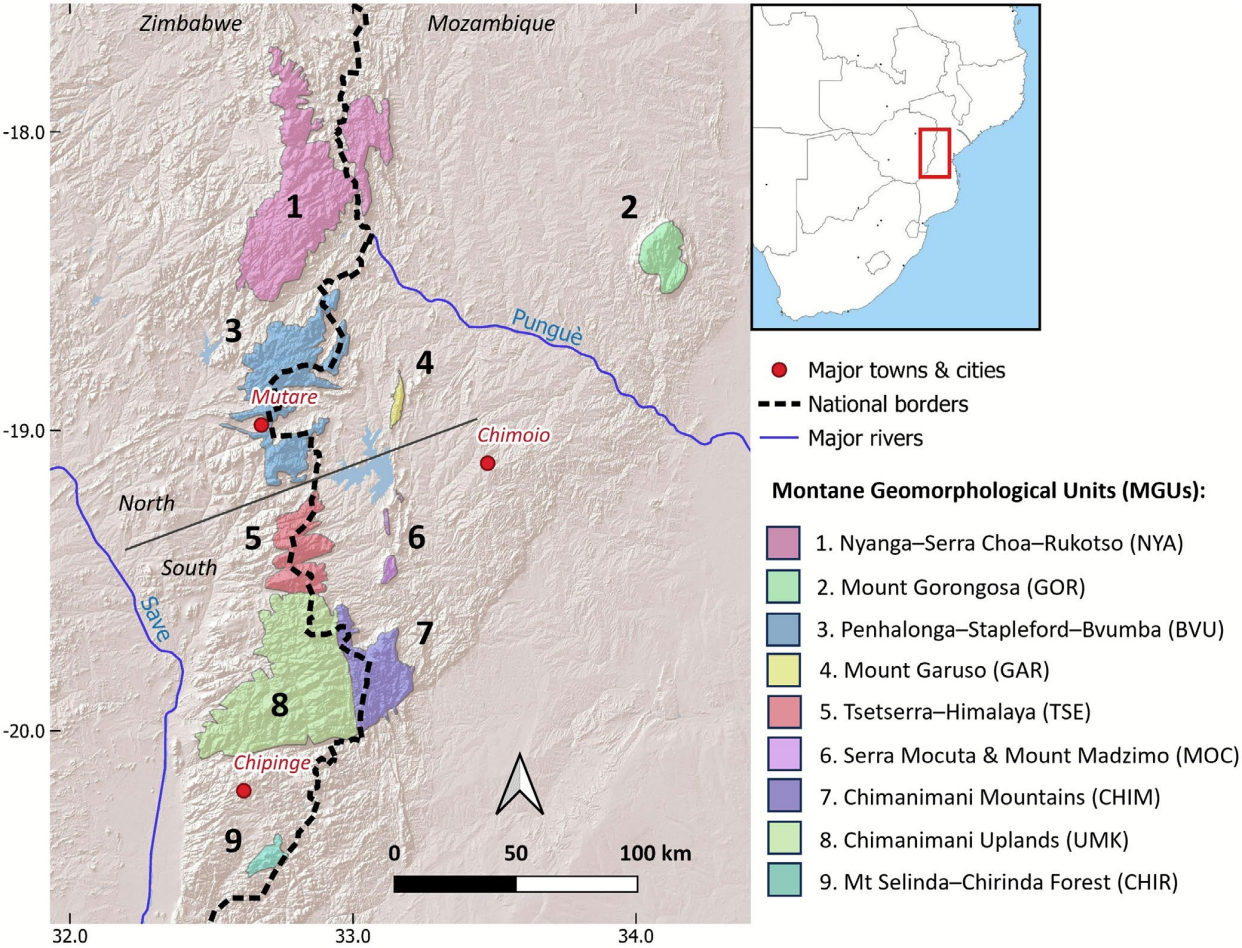
Extent of each of the nine montane geomorphological units (MGUs; see Table 1) across the Manica Highlands (Zimbabwe–Mozambique). Credit: Tom Timberlake.

**PLATE 1:**
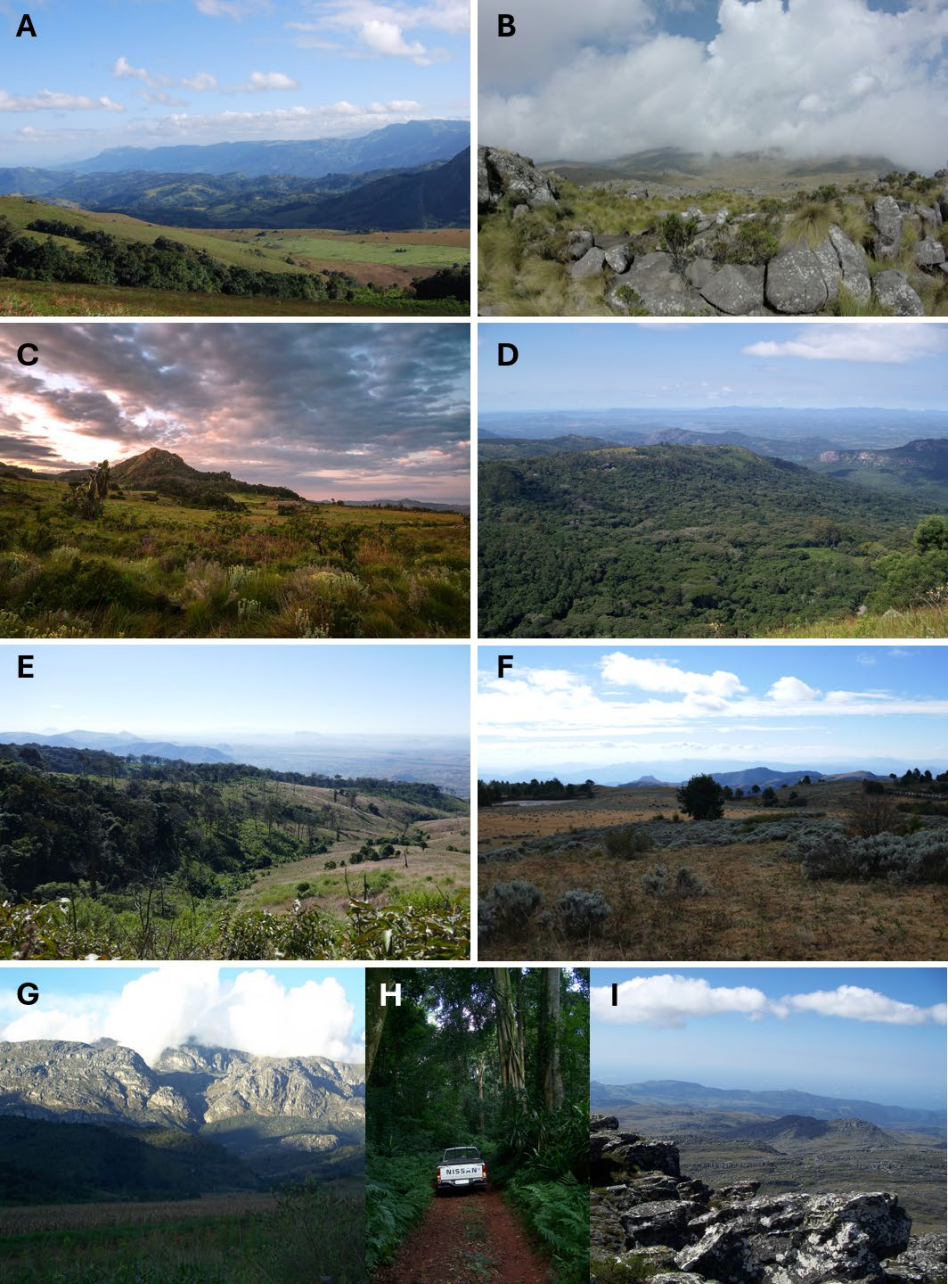
Landscapes in the Manica Highlands (Zimbabwe–Mozambique), representing the nine Montane Geomorphological Units (MGUs; see Table 1). (A) Serra Choa (Mozambique) looking towards Mount Nyangani (Zimbabwe) (MGU 1); (B) the summit of Mount Nyangani (Zimbabwe), the highest point in the Manica Highlands (2592 m) (MGU 1); (C) summit montane grassland/montane scrub on Mount Gorongosa (Mozambique) (MGU 2); (D) Bvumba, from the summit of Castleburn (Zimbabwe), looking east into Mozambique (MGU 3); (E) Mount Garuso (Mozambique) showing extensive deforestation in 2018 (MGU 4); (F) summit plateau of Tsetserra–Himalaya (Mozambique)—note the incursion of invasive alien *Pinus patula* Schltdl. & Cham. (PINACEAE) trees (MGU 5); (G) the western front of the Chimanimani Mountains (MGU 7), viewed from the Chimanimani Uplands (MGU 8) (both Zimbabwe); (H) view from within Chirinda Forest (Zimbabwe) (MGU 9); (I) the Chimanimani Mountains, looking eastwards from the summit of Mount Binga (Zimbabwe–Mozambique)—at 2436 m it is the second‐highest point in the Manica Highlands and the highest point in Mozambique (MGU 7). Credits: John Burrows (A), V. Ralph Clark (B, D, G–I), Piotr Naskrecki (C), Jo Osborne (E, F).

The Manica Highlands comprises all the montane or upland massifs associated with the Zimbabwe−Mozambique borderlands between the Zambezi and Limpopo rivers (Clark et al. 2017, 2019; Figure 1) extending from the Nyanga Mountains in the north to Chirinda Forest in the south. It includes the following localities (from north to south): Rukotso, Serra Choa, World's View–Troutbeck, Nyanga Downs, Nyanga National Park, Juliasdale, Mount Gorongosa, Mount Gorongo, Stapleford Forest Land, Penhalonga–Quinta da Fronteira, Mount Nhaungué–Garuso Forest, Cecil Kop, Bvumba–Serra Vumba, Banti Forest, Tsetserra–Himalayas, Serra Mocuta, Cashel, Rotanda, Chimanimani Mountains, the uplands of Chimanimani District–Gwendingwe, Mount Pene–Tarka–Glencoe Forest Lands and the Chirinda–Mount Selinda area in Zimbabwe (Figure 2, Plate 1).

Smaller but significant free‐standing montane areas above 1000 m elevation adjacent to the Highlands, such as Mount Gorongosa, Mount Garuso (Nhaungué), Mount Madzimba, and Serra Mocuta in Mozambique, are here included (Figure 2, Table 1). However, outlying wooded rocky inselbergs ‐at lower elevations, such as Mount Zembe and Mount Mruwere in Mozambique, have been excluded, along with the medium‐elevation Mashonaland Plateau that extends west through central Zimbabwe. From existing work (e.g., Clark et al. 2017), the latter area is known to be of low significance for endemics. The total mapped extent is around 7400 km^2^.

**TABLE 1:** The nine montane geomorphological units (MGUs) of the Manica Highlands (Zimbabwe–Mozambique), including Mount Gorongosa and significant inselbergs, together with the two sections (Northern and Southern). Area extents determined from topography and natural intervals (gaps) between MGUs.

Montane Geomorphological Unit (MGU)	Area (km^2^)	Section total (km^2^)
1. Nyanga–Serra Choa–Rukotso (NYA)	2230	Northern (MH‐N) 3870
2. Mount Gorongosa (GOR)	320	
3. Penhalonga–Stapleford–Bvumba (BVU)	1260	
4. Mount Garuso (GAR)	60	
5. Tsetserra–Himalaya (TSE)	560	Southern (MH‐S) 3530
6. Eastern outliers – Serra Mocuta & Mount Madzimo (MOC)	50	
7. Chimanimani Mountains (CHIM)	670	
8. Chimanimani Uplands (Umkondo Sandstones) (UMK)	2150	
9. Mt. Selinda–Chirinda (CHIR)	100	
Total	7400	

The great majority of the Manica Highlands lies above 1200 m elevation but, due to its asymmetrical escarpment nature (Clark et al. 2017), part of this topographically rugged terrain extends down to 400 m on the eastern Chimanimani Mountain footslopes and to 800–900 m along some other southern and eastern margins. The underlying geology across the area is predominantly syenite or granite (Stagman 1978; ING 1987) apart from the Chimanimani Mountains, which comprise Umkondo quartzites of the Frontier Series, and the Chimanimani Uplands which consist of Umkondo sandstones and dolerite (Watson 1969; Timberlake, Darbyshire, Wursten, et al. 2016). Both Mount Nyangani and Rukotso above 2000 m elevation comprise dolerite sills while Chirinda Forest lies on dolerite‐derived soils (Watson 1969; Timberlake and Shaw 1994).

Rainfall principally occurs during the warmer summer months of November to March with annual precipitation ranging from 1070 to around 3000 mm (Agritex 1989). Especially above 1700 m and on east‐facing slopes, frequent mists and low cloud (locally called *guti*) reduce evaporative stress during the cold, dry season and there can be moderate levels of winter rain (Timberlake et al. 2020). The mountains are a major source of water for two major cities and various other large population centers.

**PLATE 2:**
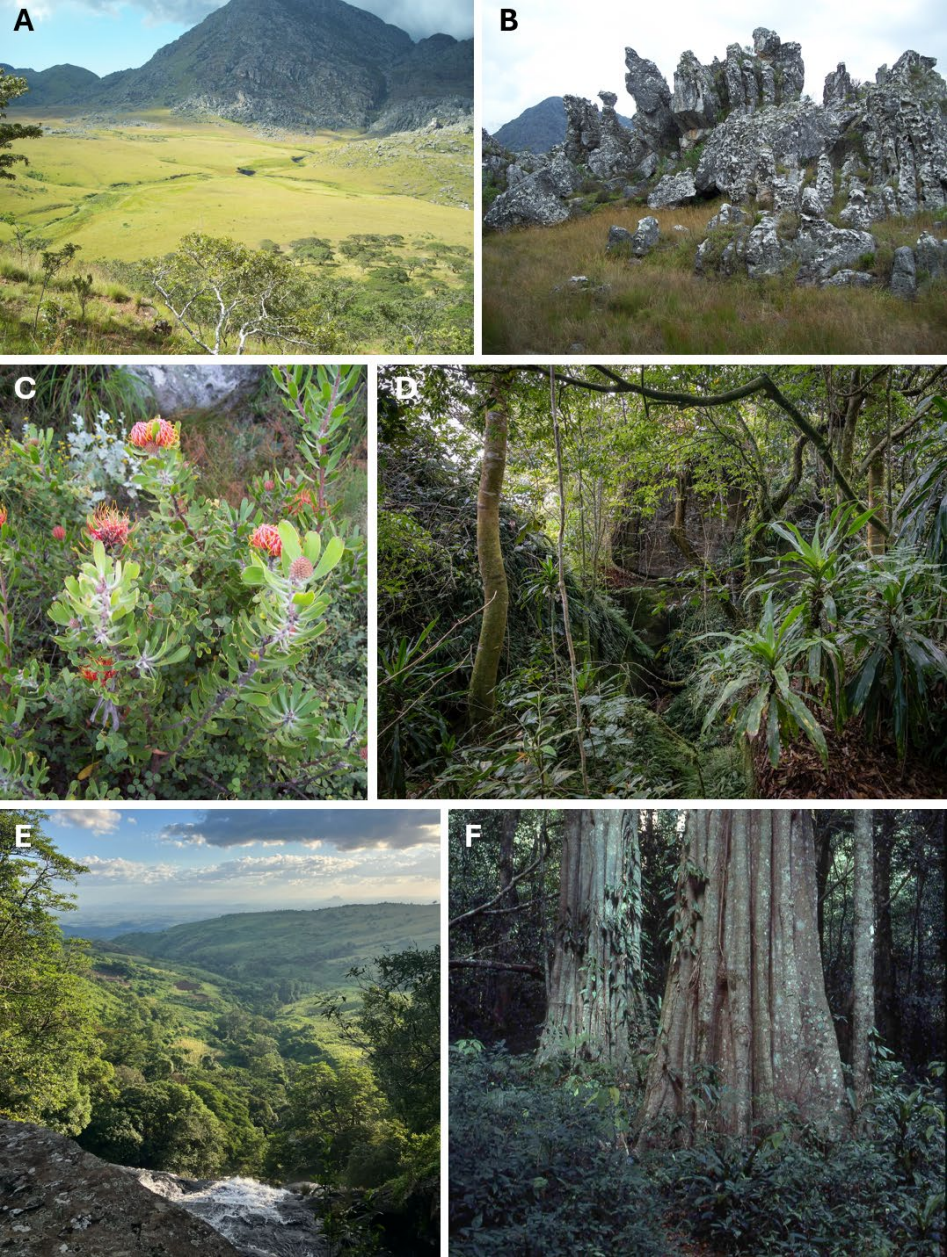
Habitats of endemic plant taxa in the Manica Highlands (Zimbabwe–Mozambique); abbreviations correlate with Table 4. (A) Bundi Valley in the Chimanimani Mountains (Zimbabwe) showing Wet Grassland/Wetland (WG/WET), Rocks & Crags (ROCK), and *Brachystegia spiciformis* Benth. (Fabaceae) savanna woodland (WL); (B) scattered large rocks (ROCK) that are part of the “Stonehenge” area in a matrix of Montane Grassland (MG), Chimanimani Mountains (Zimbabwe); (C) an example of very diverse montane scrub (MS; “fynbos”) here dominated by the near‐endemic *Leucospermum saxosum* S.Moore (Proteaceae) in the Chimanimani National Park (Zimbabwe); (D) remnant moist montane forest (MMF) on the upper slopes of Mount Gorongosa (Mozambique); (E) riverine forest (RIV) on the lower slopes of Mount Gorongosa—much of these slopes used to be covered in moist lowland forest (MLF) and woodland savanna (WL) (Mozambique); (F) moist lowland forest (LMF), showing the non‐endemic tree *Chrysophyllum gorungosanum* Engl. (Saptoceae) from Chirinda Forest. Credits: V. Ralph Clark (A–C), Piotr Naskrecki (D), Thor Kuchler (E), Phil Shaw (F).

The main vegetation types (see Plate 2) are moist miombo woodland (*Brachystegia* Benth.) and similar woodlands (e.g., *Uapaca* Baill.) at lower elevations (< 1500 m), with scrub (scattered small trees, shrubs and bracken *Pteridium aquilinum* (L.) Kuhn in a coarse grassland) higher up at 1500–1700 m. This grades into montane grassland above 1700 m (Gilliland 1938; Phipps and Goodier 1962; Wild and Barbosa 1968), while a maccia (fynbos)‐like dwarf Ericaceous and Proteaceous shrubland intermixed with bare rock found at the highest elevations of Nyanga and Chimanimani above 2000 m (Phipps and Goodier 1962; White 1978) forms part of what has been regarded as a subalpine region (Killick 1978). Numerous patches of moist evergreen forest are found on east and south‐east facing slopes, particularly at elevations above 1600 m (Müller 1999, 2006). Earlier studies have shown that there is 107 km^2^ of evergreen moist forest within the study area in Zimbabwe (Müller 2006; Timberlake et al. 2021), with a similar extent in Mozambique including semi‐deciduous forest at lower altitudes (Timberlake, Darbyshire, Cheek, et al. 2016).

In biogeographical terms, the Manica Highlands form the southern limit of the Eastern Afromontane Hotspot (Critical Ecosystem Partnership Fund 2012a, 2012b) comprising the mountains extending from Yemen through Ethiopia, East Africa and the Albertine Rift to Zimbabwe and Mozambique. An area of particular significance for biodiversity conservation, it is broadly based on White's Afromontane Floristic Region (White 1983). The Manica Highlands are also recognized as the north‐eastern terminus of the endemic‐rich southern African Great Escarpment (Clark et al. 2011a) with close biogeographical relationships to mountains south of the Limpopo River (e.g., Weimark 1941; van Wyk and Smith 2001; Hahn 2017; Clark et al. 2022).

Administratively, the Manica Highlands comprises parts of Manica Province in Zimbabwe and Manicaland Province in Mozambique. The major population centres of (from north to south) Mutare, Manica, Chimoio, Sussundenga, and Chipinge lie immediately outside the study area, although some smaller population centres such as Nyanga, Juliasdale, Penhalonga, Chimanimani town, and Mount Selinda lie inside. Land use is principally commercial and subsistence farming, forestry plantations, and formal (State) conservation. Around a third of the total extent is formally protected as National Park, National Reserve, Forest Land, or Forest Reserve, and there are many small private nature reserves. Tourism (local and international) is an important economic activity, especially in Zimbabwe.

### 1.2 Previous Botanical Studies

The existence of a distinct floristic centre in the area between the Zambezi and Limpopo Rivers was first outlined by Weimark (1941: 78) as his Inyangani Subcentre of the Cape flora (Wild 1956). Later, this was better defined by Frank White as the Chimanimani Regional Mountain System (White 1978), one of seven blocks within his Afromontane archipelago‐like regional centre of endemism spanning the continent north to south (White 1983). The existence of two associated centres of endemism—the Chimanimani Mountains (Af79) and Nyanga (Af80)—was mentioned by Davis et al. (1994) in their study on centres of plant diversity, but with only brief descriptions, while Küper et al. (2004) regard their Chimanimani Centre, incorporating both the Chimanimani and Nyanga Mountains, to be a priority for conservation action. In his study on the phytogeography of south‐central Africa, Wild (1968: 209) highlighted the significance of this montane area and he earlier (Wild 1964) provided the first detailed description of the endemics of the Chimanimani Mountains, suggesting that the disjunction between the Manica Highlands, mountains south of the Limpopo River, and Mount Mulanje and others in Malawi to the north may date from the Tertiary Period (Wild 1964: 132) around 50 mya. However, more recent biogeographical findings would suggest this age seems unlikely and floristic separation may have only occurred around 5 million years ago in the late Miocene (Linder 2014), with ancestral lineages emerging in the Miocene (20 mya).

Although the Manica Highlands have been the subject of much botanical exploration and plant diversity documentation over the last 100 years (see Macedo 1970; Clark et al. 2017; Wursten et al. 2017; Timberlake et al. 2020), a synthesis on endemism remains incomplete. For example, van Wyk and Smith (2001) in their comprehensive description of the Chimanimani–Nyanga Centre only give an estimate of around 100 endemic taxa, and Mapaura (2002) only considers endemics on the Zimbabwe side. Wild (1964) and Wursten et al. (2017) discuss endemics from just the Chimanimani Mountains, while Clark et al. (2017) and Timberlake et al. (2020) cover just the Nyanga and Bvumba components, respectively. There is no detailed vegetation study of the Manica Highlands as a whole, although low‐detail maps exist for the Chimanimani Mountains (Goodier and Phipps 1962) and Mount Gorongosa (Macedo 1970).

This study comes as part of a steadily growing series of papers on plant diversity and endemism across the mountains of southern Africa, including the Nyika Plateau (Burrows and Willis 2005), Mount Mulanje (Strugnell 2006), Mount Namuli (Timberlake et al. 2009; Timberlake 2021), Mount Mabu (Timberlake et al. 2012; Bayliss et al. 2014), the inselbergs of northern Mozambique and southern Malawi (Bayliss et al. 2024), Mount Gorongosa (Macedo 1970; Müller et al. 2012), Nyanga (Clark et al. 2017), the Bvumba (Timberlake et al. 2020), Chimanimani (Goodier and Phipps 1961; Phipps and Goodier 1962; Timberlake, Darbyshire, Wursten, et al. 2016a; Wursten et al. 2017), Chirinda (Drummond and Mapaure 1994; Timberlake and Shaw 1994), Soutpansberg (Hahn 2017), the Limpopo–Mpumalanga–Eswatini Escarpment (henceforth LMEE; Clark et al. 2022), and the Maloti–Drakensberg (=Drakensberg Mountain Centre; Carbutt 2019), along with other southern African mountains including those in the Cape provinces (Clark et al. 2009, 2011b, 2014), Namibia (Craven and Kolberg 2023; De Cauwer et al. 2023), and Angola (Mendelsohn et al. 2023).

## 2 Methods

### 2.1 Definition of Study Area and Determination of Montane Geomorphological Units

For the purposes of capturing as many endemics as possible across these borderlands, we initially used the broad orographic delimitation of Clark et al. (2017, 2019) as the study area, with the addition of significant nearby inselbergs over 1000 m elevation (viz. Mounts Gorongosa, Nhaungue, Madzimba, and Serra Mocuta), giving a total extent of around 8200 km^2^. As existing knowledge of the area (van Wyk and Smith 2001) indicates that most endemics are higher elevation species, this was considered reasonable and follows similar approaches elsewhere in southern Africa, for example the Sneeuberg Centre (Clark et al. 2009); the Great Winterberg–Amatholes (Clark et al. 2014); the Drakensberg Mountain Centre (Carbutt 2019); and the LMEE (Clark et al. 2022). However, to better define a Manica Highlands Centre, we have been more restrictive and, along with elevation and topography, were guided by the recorded distributions of endemic taxa occurring within the area and the main montane habitats. Google Earth imagery was used for the delineation. The present mapped extent (Figure 2) is thus smaller (7400 km^2^) than the broader orographic Manica Highlands area. For example, a large extent of the gently undulating uplands in Chipinge District in the south was omitted from the original delineation as no endemics were recorded from there and the area is not montane.

Following Clark et al. (2017), we subdivided the Manica Highlands into nine montane geomorphological units (MGUs) based on two physical geographic criteria—(i) discrete sections divided by clear low‐elevation intervals, and (ii) sections that have clear distinctiveness within a larger orographic unit through strong internal geological variation (see Figure 2). From north to south these are: Nyanga (including Rukotso and Serra Choa); Mount Gorongosa; Penhalonga–Stapleford–Bvumba; Mount Garuso; Tsetserra–Himalaya (including Banti and Rotanda); Serra Mocuta and Mount Madzimo; the core Chimanimani Mountains; other uplands of the Chimanimani District (including various State Forest Lands); and the separate Chirinda Forest–Mount Selinda. Their extents are given in Table 1.

As some endemic taxa are known to occur across a number of MGUs, we also divided the whole Manica Highlands area into two approximately equal‐sized sections—a Northern section and a Southern section (Figure 2) using the low‐elevation Burma Valley just south of the Bvumba as a natural divide. The Northern section (including Mount Gorongosa and the Mount Garuso area) totals 3870 km^2^ while the area south of the Burma Valley (including Serra Mocuta and Mount Madzimo) is 3530 km^2^. Patterns of endemism were determined by calculating the number of listed endemics occurring in each MGU or section, as well as those across these.

### 2.2 Species Selection

An initial list of candidate endemic taxa was compiled from existing literature on the region (e.g., Mapaura 2002; Mapaura and Timberlake 2002; Clark et al. 2017; Wursten et al. 2017; Darbyshire et al. 2019; Timberlake et al. 2020) supplemented by published volumes of *Flora Zambesiaca* (1960–continuing) and recent descriptions of novel species. Relevant information on location, elevation and habitat (if indicated) from labels of over 2500 confirmed herbarium specimens of these taxa at the Royal Botanic Gardens, Kew (K) and Harare (SRGH) herbaria was then noted along with any ancillary ecological data. However, such data are known to be often incomplete or unreliable, particularly for older records. Specimen label data were supplemented by georeferenced records and observations from regional literature and the Flora of Zimbabwe and Flora of Mozambique websites (Hyde et al. 2024a, 2024b).

Criteria used for confirmation of endemic status were both taxonomic and spatial. Taxonomically, candidates had to be recognized taxa of vascular plants according to current literature (we followed the African Plant Database website https://africanplantdatabase.ch/en, generally regarded as the standard for the region) and only formally recognized species, subspecies and varieties were included. Published “forma” and unpublished taxa were not included unless ancillary information (RBG Kew specialists, pers. comm.) indicated that they are indeed distinct and will later be formally described, for example, *Rytigynia* sp. E (Rubiaceae) of *Flora Zambesiaca* and *Indigofera* sp. near *I. chimanimaniensis* Schrire (Fabaceae). Indistinct varieties were combined under their subspecies or removed. Nomenclature, families and authorities follow those in current use in the African Plant Database website (https://africanplantdatabase.ch/en, March 2025).

Spatially, taxa were determined as being strictly endemic based on their exclusive confinement to the study area based on herbarium collections, *Flora Zambesiaca*, and later literature sources. Taxa with a portion of their distribution some distance away, or which were confined to nearby isolated non‐montane inselbergs such as Mount Zembe and Mount Mruwere, were excluded.

Near‐endemics were defined as taxa where, in addition to the main populations being found within the study area, records are known from other mountains or inselbergs. Included in this category were taxa with isolated occurrences on mountains in South Africa some 400 km south (i.e., *Leucospermum saxosum* S.Moore and *Protea dracomontana* Beard, both Proteaceae) or on Mount Mabu or other mountains up to 500 km north‐east in Mozambique (see Timberlake et al. 2012). Near‐endemics are listed in Table A2 in Appendix 1, while excluded taxa are listed in Table A3 in Appendix 1 together with reasons for exclusion. Only full endemics (Table A1 in Appendix 1) were used in the analyses.

### 2.3 Habitats

All stated habitats shown on herbarium specimens, in other records and personal observations were grouped into nine categories or habitat groups: rock outcrops or crags (ROCK); montane grassland (MG); montane scrub including fynbos‐like vegetation (MS); forest margins (FM); montane moist forest (MMF); lowland moist forest (including late‐deciduous forest; LMF); woodland (principally miombo dominated by *Brachystegia spiciformis* Benth.; WL); wetland/seepages (WET); and riverine forest (RIV)—see Table 4. As some species occur in more than one habitat and habitats stated on older herbarium labels can be misleading, a simplified amalgamated system was then used to focus primarily on open and closed habitats—Open Montane (ROCK + MG + MS + WET) and Moist Forest (LMF + MMF + FM + RIV), plus Woodland (including transformed non‐montane vegetation such as secondary grassland and scrub; WL). Each taxon was assigned to one of these three amalgamated units based on the categories shown in Table A1 in Appendix 1 or, in the case of multiple habitats, the main group to which records were allocated.

Determination of the extent of primary habitats was not possible as there are no vegetation maps available for the Manica Highlands as a whole and there is a lot of settled land, agricultural fields and plantation; in addition, boundaries between habitats are often not clear. Therefore, Google Earth imagery, most of it dating from 2023, was used to provide rough assessments of (a) the extent of montane grassland and rocky outcrops, and (b) the extent of moist forest and dense woody vegetation, including late‐deciduous forest (*sensu* Timberlake, Darbyshire, Cheek, et al. 2016). The remaining extent includes settled land, cultivation, commercial forest plantation, secondary grassland or scrub and woodland dominated by *Brachystegia* and *Uapaca*.

### 2.4 Life‐Forms

Using descriptions in regional floras and similar publications, species' lifeforms were classified simply into herbs (annual herb, perennial herb, graminoid, succulent, epiphyte, geophyte with bulb or corm), trees, and shrubs (including climbers and holo‐parasites), although some taxa can occur in two groups.

### 2.5 Substrate

Apart from taxa recorded as being primarily on quartzite in the Chimanimani area, it was not feasible to reliably allocate records to geological substrate as information on herbarium labels is often missing or uncertain. However, most other records are probably from syenite or granite substrates, apart from those at higher elevations on Nyanga or Rukotso on dolerite sills and those in the Chimanimani Uplands and Chirinda Forest on Umkondo sandstones.

### 2.6 Elevation Partitioning

The elevation range of endemics was noted from source literature, specimen labels and georeferenced records, although these are only approximate for older specimens. Particular attention was paid to taxa occurring above 2000 m elevation.

### 2.7 Conservation Status

For the endemic taxa that have been assessed, the current conservation status for each taxon was taken from the published IUCN Red List, version 2024–2 (IUCN 2024), along with two completed but as yet unpublished assessments (*Protea caffra* Meisn. subsp. *gazensis* (Beard) Chisumpa & Brummitt and *Synsepalum chimanimani* S.Rokni & I.Darbysh.).

### 2.8 Analyses

Determination of endemic richness by montane geomorphological unit and comparison of Manica Highlands endemic richness with other summer rainfall southern African mountain systems was done using simple regression analyses in Microsoft Excel using log‐transformed values for area and endemic diversity. Non‐independent variables in the regressions for MGUs (e.g., Northern and Southern sections) and centers of endemism (i.e., the Wolkberg embedded within the larger LMEE) were excluded from the regression line equations and manually inserted separately into the graphics in red for comparative purposes only.

## 3 Results

### 3.1 Endemic Taxa and Distribution

**PLATE 3:**
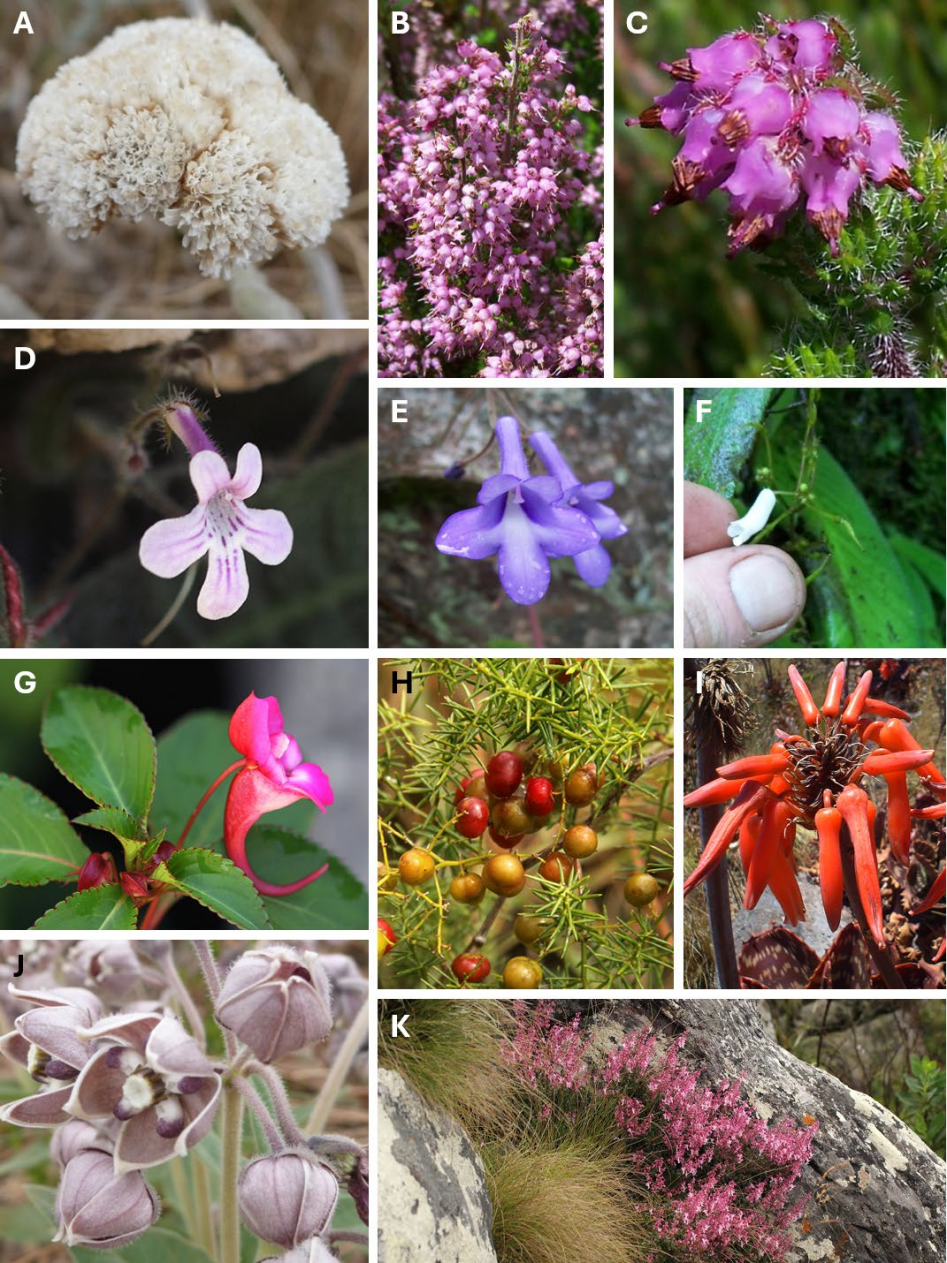
A selection of representatives of the 216 plant taxa endemic to the Manica Highlands (Zimbabwe–Mozambique). (A) *Helichrysum acervatum* S.Moore (Asteraceae), endemic to the main spine of the Manica Highlands; (B) *Erica pleiotricha* var. *blaerioides* (Wild) R.Ross and (C) *E. lanceolifera* S.Moore (Ericaceae) are both endemic to the Southern section where they are typical in montane scrub (‘fynbos’); there are nine *Streptocarpus* (Gesneriaceae) endemic to the Manica Highlands of which (D) *S. hirticapsa* B.L.Burtt is confined to the Southern section, while (E) *S. michelmorei* B.L.Burtt and (F) *S. umtaliensis* B.L.Burtt are found down the main spine of the whole Manica Highlands; (G) *Impatiens salpinx* G.M.Schulze & Launert (Balsamaceae) is endemic to forests in the Chimanimani Mountains while (H) *Asparagus chimanimanensis* Sebsebe (Asparagaceae) is endemic to quartzite rock outcrops in the Chimanimani Mountains; one of 15 endemic *Aloe* taxa (Asphodelaceae) endemic to the Manica Highlands (I) *Aloe collina* S.Carter is one of the more widespread species; (J) *Asclepias fimbriata* Weim. (Apocynaceae) is also a widespread endemic in the Manica Highlands, as is (K) *Plectranthus chimanimanensis* S.Moore (Lamiaceae). Credits: V. Ralph Clark (A–F, J), Bart Wursten (G, H, K).

There are 216 vascular plant taxa strictly endemic to the study area (Table 2, Table A1 in Appendix 1, Plate 3) plus 13 near‐endemics (Table A2 in Appendix 1). Strict endemics comprise 55 monocotyledons, 160 dicotyledons, and one gymnosperm (the cycad *Encephalartos chimanimaniensis* R.A.Dyer & I.Verd.), in 51 families; there are no endemic pteridophytes. There is only one endemic genus (*Oligophyton* H.P.Linder; Orchidaceae) and no endemic family. The 10 largest families are Fabaceae *sensu lato* (25 taxa), Asteraceae (19), Rubiaceae and Orchidaceae (both 18), Asphodelaceae (15), Apocynaceae *sensu lato* (12), Lamiaceae and Gesneriaceae (both 8), Iridaceae (7), and Scrophulariaceae (6). The most endemic‐rich genera are *Aloe* (15 taxa), *Streptocarpus* (8), *Helichrysum* and *Tephrosia* (both 6), *Aeschynomene* and *Indigofera* (both 5).

**TABLE 2:** Plant taxa endemic to the Manica Highlands (Zimbabwe–Mozambique). Taxa arranged alphabetically under family; nomenclature, family and authority follows that in current use in the African Plant Database website (https://africanplantdatabase.ch/en, March 2025).

Endemic taxon	IUCN Red Listing	MGU endemic to	Lifeform	Primary habitat	> 2,000 m
Gymnosperms					
Zamiaceae					
*Encephalartos chimanimaniensis* R.A.Dyer and I.Verd.	EN	MH‐S	s	**OM**	
Monocotyledons					
Amaryllidaceae					
*Scadoxus pole‐evansii* (Oberm.) Friis and Nordal	NT	NYA	h(geo)	**FOR**	
*Tulbaghia friesii* Suess.	LC	NYA	h(geo)	**OM**	x
Asparagaceae					
*Asparagus chimanimanensis* Sebsebe	LC	CHIM	s	**OM**	x
*Chlorophytum pygmaeum* (Weim.) Kativu subsp. *rhodesianum* (Rendle) Kativu		MH‐S	h(geo)	**OM**	
*Eriospermum cecilii* Baker	LC	NYA	h(geo)	**OM**	xx
*Eriospermum mackenii* (Hook.f.) Baker subsp. *phippsii* (Wild) P.L.Perry		CHIM	h(geo)	**OM**	
*Ledebouria ciliata* (Baker) Stedje		NYA	h(geo)	**OM**	x
Asphodelaceae					
*Aloe ballii* Reynolds var. *ballii*	VU	CHIM low elev	h(suc)	**OM**	
*Aloe ballii* Reynolds var. *makurupiniensis* Ellert	VU	CHIM low elev	h(suc)	**OM**	
*Aloe cameronii* Hemsl. var. *bondana* Reynolds	LC	MH	h(suc)	**OM**	x
*Aloe cannellii* L.C.Leach	LC	MH	h(suc)	**OM**	
*Aloe collina* S.Carter	VU	NYA	h(suc)	**OM**	xx
*Aloe hazeliana* Reynolds var. *hazeliana*	LC	CHIM	h(suc)	**OM**	x
*Aloe hazeliana* Reynolds var. *howmanii* (Reynolds) S.Carter	LC	CHIM	h(suc)	**OM**	x
*Aloe inyangensis* Christian var. *inyangensis*	LC	NYA	h(suc)	**OM**	x
*Aloe inyangensis* Christian var. *kimberleyana* S.Carter	LC	MH	h(suc)	**OM**	
*Aloe munchii* Christian	LC	CHIM	s(suc)	**OM**	x
*Aloe musapana* Reynolds	VU	MH‐S	h(suc)	**OM**	
*Aloe plowesii* Reynolds	VU	CHIM	h(suc)	**OM**	
*Aloe rhodesiana* Rendle	VU	MH	h(suc)	**OM**	x
*Aloe swynnertonii* Rendle		MH	h(suc)	**OM**	
*Aloe wildii* (Reynolds) Reynolds	LC	CHIM	h(suc)	**OM**	
Commelinaceae					
*Cyanotis chimanimaniensis* Faden INED.		CHIM	h(p)	**OM**	
Eriocaulaceae					
*Mesanthemum africanum* Moldenke	LC	CHIM	h(p)	**OM**	x
Iridaceae					
*Dierama inyangense* Hilliard	EN	MH	h(geo)	**OM**	x
*Dierama plowesii* Hilliard	VU	MH	h(geo)	**OM**	x
*Gladiolus flavoviridis* Goldblatt	VU	BVU	h(geo)	**OM**	
*Gladiolus juncifolius* Goldblatt		MH	h(geo)	**OM**	x
*Gladiolus zimbabweensis* Goldblatt	VU	MH	h(geo)	**OM**	x
*Hesperantha ballii* Wild	LC	CHIM	h(geo)	**OM**	x
*Moraea inyangani* Goldblatt	LC	NYA	h(geo)	**OM**	xx
Orchidaceae					
*Aerangis verdickii* (De Wild.) Schltr. var. *rusituensis* (Fibeck and Dare) la Croix and P.J.Cribb		CHIM low elev	h(epi)	**WL**	
*Aeranthes africana* J.L.Stewart	CR	BVU	h(epi)	**FOR**	
*Aeranthes parkesii* G.Will.	CR	NYA low elev	h(epi)	**FOR**	
*Angraecum chimanimaniense* G.Will.		CHIM	h(epi)	**FOR**	
*Cynorkis anisoloba* Summerh.	LC	MH	h(geo)	**OM**	x
*Disa chimanimaniensis* (H.P.Linder) H.P.Linder	LC	CHIM	h(geo)	**OM**	
*Disa zimbabweensis* H.P.Linder	VU	MH	h(geo)	**OM**	
*Liparis chimanimaniensis* G.Will.		MH	h(geo)	**OM**	xx
*Neobolusia ciliata* Summerh.	EN	MH	h(geo)	**OM**	x
*Neobolusia stolzii* Schltr. var. *glabripetala* Summerh.	VU	NYA	h(geo)	**OM**	x
*Oligophyton drummondii* H.P.Linder and G.Will.	LC	CHIM	h(geo)	**OM**	
*Polystachya subumbellata* P.J.Cribb and Podz.	LC	MH	h(epi)	**FOR**	
*Satyrium flavum* la Croix	LC	MH	h(geo)	**OM**	x
*Satyrium hallackii* Bolus subsp. *ballii* (van der Niet and P.J.Cribb) van der Niet and P.J.Cribb		MH	h(geo)	**OM**	
*Satyrium mirum* Summerh.		MH‐S	h(geo)	**OM**	xx
*Schizochilus calcaratus* P.J.Cribb and la Croix		CHIM	h(geo)	**OM**	
*Schizochilus cecilii* Rolfe subsp. *cecilii*	LC	NYA	h(geo)	**OM**	xx
*Schizochilus lepidus* Summerh.	VU	MH‐S	h(geo)	**OM**	x
Poaceae					
*Danthoniopsis chimanimaniensis* (J.B.Phipps) Clayton	EN	CHIM	h(gr)	**OM**	
*Digitaria fuscopilosa* Goetgh.	DD	TSE	h(gr)	**OM**	
*Eragrostis desolata* Launert	LC	CHIM	h(gr)	**OM**	
Restionaceae			h(gr)		
*Platycaulos quartziticola* (H.P.Linder) H.P.Linder and C.R.Hardy	LC	CHIM	h(gr)	**OM**	x
Velloziaceae					
*Xerophyta argentea* (Wild) L.B.Sm. and Ayensu	LC	MH‐S	s, h(p)	**OM**	x
Xyridaceae					
*Xyris asterotricha* Lock	VU	CHIM	h(p)	**OM**	
Dicotyledons					
Acanthaceae					
*Barleria fissimuroides* I.Darbysh.	EN	BVU (Mutare)	s	**WL**	
*Dicliptera nyangana* I.Darbysh.	LC	NYA	h(p)	**OM**	
*Justicia subcordatifolia* Vollesen and I.Darbysh.		MH‐N	h(p)	**OM**	x
Apiaceae					
*Afrosciadium rhodesicum* (Cannon) P.J.D.Winter	VU	MH	h(p)	**OM**	x
*Centella obtriangularis* Cannon	VU	CHIM	h(p)	**OM**	
*Diplolophium buchananii* (Oliv.) Norman subsp. *swynnertonii* (Baker f.) Cannon	LC	MH	h(p)	**OM**	
Apocynaceae					
*Asclepias cucullata* (Schltr.) Schltr. subsp. *scabrifolia* (S.Moore) Goyder		MH	h(geo)	**OM**	x
*Asclepias fimbriata* Weim.	LC	MH	h(geo)	**OM**	x
*Asclepias graminifolia* (Wild) Goyder	LC	CHIM	h(geo)	**OM**	xx
*Aspidoglossum glabellum* Kupicha	EN	MH‐S	h(geo)	**OM**	
*Aspidoglossum rhodesicum* (Weim.) Kupicha		NYA	h(geo)	**OM**	x
*Ceropegia chimanimaniensis* M.G.Gilbert	LC	CHIM	h(geo)	**OM**	
*Ceropegia spatuliloba* M.G.Gilbert	CR	TSE	h(geo)	**OM**	xx
*Huernia hislopii* Turrill subsp. *cashelensis* (L.C.Leach and Plowes) Bruyns	NT	MH‐S	h(suc)	**WL**	
*Marsdenia chirindensis* Goyder		CHIR	cl	**FOR**	
*Raphionacme chimanimania* Venter and R.L.Verh.	EN	CHIM	h(geo)	**OM**	
*Sisyranthus rhodesicus* Weim.		MH‐N	h(p)	**OM**	xx
*Vincetoxicum monticola* Goyder		MH	h(geo)	**FOR**	
Asteraceae					
*Afroaster chimanimaniensis* (W.Lippert) J.C.Manning and Goldblatt	DD	CHIM	h(p)	**OM**	
*Anisopappus paucidentatus* Wild	LC	CHIM	h(p)	**OM**	x
*Athrixia fontinalis* Wild		MH	h(p)	**OM**	x
*Baccharoides calvoana* (Hook.f.) Isawumi, El‐Ghazaly and B.Nord. subsp. *meridionalis* (Wild) Isuwami, El‐Ghazaly and B.Nord.		MH	h(p), s	**OM**	
*Calomeria africana* (S.Moore) Heine	LC	CHIM	h(p), s	**OM**	x
*Cineraria pulchra* Cron		MH	h(p), s	**OM**	
*Helichrysum acervatum* S.Moore		MH	h(p)	**OM**	x
*Helichrysum chasei* Wild		MH	h(p)	**OM**	
*Helichrysum inyangense* Wild	LC	NYA	h(p)	**OM**	x
*Helichrysum maestum* Wild		CHIM	h(p)	**OM**	xx
*Helichrysum rhodellum* Wild		MH‐S	h(p)	**OM**	
*Helichrysum spenceranum* Wild	LC	CHIM	h(p)	**OM**	x
*Inulanthera nuda* Källersjö	LC	NYA	s	**OM**	xx
*Kleinia chimanimaniensis* van Jaarsv.		MH	h(suc)	**OM**	
*Lopholaena brickellioides* S.Moore		MH	s, t	**OM**	
*Schistostephium oxylobum* S.Moore	VU	MH	h(p), s	**OM**	x
*Senecio aetfatensis* B.Nord.	LC	CHIM	h(p)	**OM**	
*Vernonia muelleri* Wild subsp. *muelleri*	NT	CHIM	s	**FOR**	
*Vernonia nepetifolia* Wild	LC	CHIM	s	**OM**	x
Balsaminaceae					
*Impatiens cecilii* N.E.Br. subsp. *cecilii*		MH	h(p)	**FOR**	
*Impatiens salpinx* G.M.Schulze and Launert	NT	CHIM	h(p)	**OM**	
*Impatiens wuerstenii* S.B.Janssens and Dessein	VU	GOR	h(p)	**OM**	
Boraginaceae					
*Cynoglossum inyangense* E.S.Martins	VU	NYA	h(p)	**OM**	xx
*Cynoglossum wildii* E.S.Martins		MH‐S	h(p)	**FOR**	
Brassicaceae					
*Lepidium inyangense* Jonsell	DD	NYA	h(a)	**OM**	
Campanulaceae					
*Cyphia alba* N.E.Br.	LC	MH	h(cl)	**OM**	x
*Lobelia cobaltica* S.Moore	LC	CHIM	h(a)	**OM**	x
*Wahlenbergia subaphylla* (Baker) Thulin subsp. *scoparia* (Wild) Thulin		MH‐S	h(p)	**OM**	x
Caprifoliaceae					
*Pterocephalus centennii* M.J.Cannon	CR	TSE	s	**FOR**	x
Caryophyllaceae					
*Dianthus chimanimaniensis* S.S.Hooper	VU	CHIM	h(p)	**OM**	
Celastraceae					
*Maytenus chasei* N.Robson	NT	MH	s, t	**FOR**	
Crassulaceae					
*Crassula alticola* R.Fern.	LC	MH	h(suc)	**OM**	x
*Kalanchoe velutina* Wwelw. subsp. *chimanimaniensis* (R.Fern.) R.Fern.		CHIM	h(suc)	**OM**	
Ericaceae					
*Erica lanceolifera* S.Moore	VU	MH‐S	s	**OM**	x
*Erica pleiotricha* S.Moore var. *blaerioides* (Wild) R.Ross	NT	MH‐S	s	**OM**	x
*Erica pleiotricha* S.Moore var. *pleiotricha*	VU	MH‐S	s	**OM**	x
*Erica wildii* Brenan	LC	CHIM	h(p), s	**OM**	x
Euphorbiaceae					
*Euphorbia citrina* S.Carter		MH	s	**OM**	x
*Euphorbia crebrifolia* S.Carter	LC	MH	h(p)	**OM**	x
*Euphorbia depauperata* A.Rich. var. *tsetserrensis* S.Carter		TSE	h(p)	**OM**	x
*Euphorbia rugosiflora* L.C.Leach	EN	CHIM	s	**OM**	
*Necepsia castaneifolia* (Baill.) Bouchat and J.Léonard subsp. *chirindica* (Radcl.‐Sm.) Bouchart and J.Léonard	EN	CHIM	t	**FOR**	
Fabaceae					
*Aeschynomene aphylla* Wild	VU	CHIM	s	**OM**	
*Aeschynomene chimanimaniensis* Verdc.	LC	CHIM	s	**OM**	
*Aeschynomene gazensis* Baker	EN	UMK	s	**OM**	
*Aeschynomene grandistipulata* Harms	LC	CHIM	s	**OM**	x
*Aeschynomene inyangensis* Willd.	LC	MH	s	**OM**	x
*Crotalaria insignis* Polhill	VU	MH	s	**OM**	
*Crotalaria inyangensis* Polhill	LC	MH	h(p)	**OM**	xx
*Crotalaria phylicoides* Wild	LC	CHIM	h(p), s	**OM**	x
*Indigofera cecilii* N.E.Br.		MH	h(p), s	**OM**	x
*Indigofera chimanimaniensis* Schrire	EN	UMK	h(p)	**OM**	
*Indigofera longipedicellata* J.B.Gillett	LC	NYA	h(p)	**OM**	xx
*Indigofera* sp. near *I. chimanimaniensis* Schrire		CHIM	h(p)	**OM**	xx
*Indigofera vicioides* Jaub. and Spach subsp. *excelsa* Schrire		MH	h(p), s	**OM**	x
*Lotus wildii* J.B.Gillett		MH	h(p), s	**OM**	x
*Otholobium foliosum* (Oliv.) C.H.Stirt. subsp. *gazense* (Baker f.) Verdc.		CHIM	s	**WL**	x
*Pearsonia mesopontica* Polhill	LC	MH‐S	h(p)	**OM**	
*Rhynchosia chimanimaniensis* Verdc.	EN	MH‐S	h(p), s	**OM**	
*Rhynchosia stipata* Meikle	LC	CHIM	h(cl)	**OM**	x
*Rhynchosia swynnertonii* Baker f.	LC	MH	h(cl)	**OM**	x
*Tephrosia chimanimaniana* Brummitt	LC	MH‐S	s	**OM**	
*Tephrosia festina* Brummitt		MH	s	**OM**	
*Tephrosia longipes* Meisn. var. *drummondii* (Brummitt) Brummitt		MH‐S	h(p)	**OM**	
*Tephrosia longipes* Meisn. var. *swynnertonii* (Baker f.) Brummitt		MH	h(p), s	**WL**	
*Tephrosia montana* Brummitt		MH	s	**OM**	
*Tephrosia praecana* Brummitt	VU	MH‐S	s, t	**FOR**	
Geraniaceae					
*Geranium exellii* J.R.Laundon	EN	TSE	h(p)	**OM**	xx
*Pelargonium mossambicense* Engl.		MH	h(p)	**OM**	xx
Gesneriaceae					
*Streptocarpus acicularis* I.Darbysh. and Massingue	CR	CHIM low elev	h(p)	**FOR**	
*Streptocarpus brachynema* Hilliard and B.L.Burtt	EN	GOR	h(p)	**FOR**	
*Streptocarpus cyanandrus* B.L.Burtt	LC	NYA	h(p)	**OM**	xx
*Streptocarpus grandis* N.E.Br. subsp. *septentrionalis* Hilliard and B.L.Burtt		MH‐S	h(p)	**OM**	
*Streptocarpus hirticapsa* B.L.Burtt	VU	MH‐S	h(p)	**OM**	
*Streptocarpus michelmorei* B.L.Burtt		MH	h(p)	**FOR**	
*Streptocarpus montis‐bingae* Hilliard and B.L.Burtt	DD	CHIM	h(p)	**OM**	xx
*Streptocarpus umtaliensis* B.L.Burtt	LC	MH	h(p)	**FOR**	x
Lamiaceae					
*Aeollanthus viscosus* Ryding	LC	CHIM	s	**OM**	
*Coleus caudatus* (S.Moore) E.Downes and I.Darbysh.	LC	CHIM	h(p)	**OM**	
*Coleus sessilifolius* (A.J.Paton) A.J.Paton		MH	h(p)	**OM**	x
*Plectranthus chimanimanensis* S.Moore	LC	MH	h(p), s	**OM**	x
*Rotheca verdcourtii* (R.Fern.) R.Fern.	LC	MH‐N	s, t	**FOR**	x
*Syncolostemon flabellifolius* (S.Moore) A.J.Paton	LC	CHIM	s, t	**OM**	x
*Syncolostemon oritrephes* (Wild) D.F.Otieno	VU	CHIM	h(p), s	**OM**	
*Syncolostemon ornatus* (S.Moore) D.F.Otieno	VU	MH‐S	s	**OM**	x
Linderniaceae					
*Crepidorhopalon flavus* (S.Moore) I.Darbysh. and Eb.Fisch.	VU	MH‐S	h(p)	**WL**	
Loranthaceae					
*Agelanthus lancifolius* Polhill and Wiens		MH‐S	s(par)	**FOR**	
*Englerina oedostemon* (Danser) Polhill and Wiens		MH	s(par)	**FOR**	
*Englerina swynnertonii* (Sprague) Polhill and Wiens		MH‐S	s(par)	**FOR**	
Melastomataceae					
*Dissotis swynnertonii* (Baker f.) A.Fern. and R.Fern.	VU	CHIM	s	**OM**	
*Rosettea pulchra* (A.Fern. and R.Fern.) Ver.‐Lib. and G.Kadereit	VU	CHIM	h(p), s	**OM**	
Melianthaceae					
*Bersama swynnertonii* Baker f.	LC	MH	s, t	**OM**	
Moraceae					
*Ficus muelleriana* C.C.Berg	EN	CHIM low elev	s	**OM**	
Myricaceae					
*Morella chimanimaniana* Verdc.and Polhill	EN	CHIM	s	**OM**	x
Oleaceae					
*Olea chimanimani* Kupicha	LC	CHIM	s, t	**OM**	
Orobanchaceae					
*Buchnera chimanimaniensis* Philcox	LC	MH‐S	h(a)	**OM**	
*Buchnera subglabra* Philcox	VU	CHIM	h(a)	**OM**	x
Passifloraceae					
*Basananthe parvifolia* (Baker f.) J.J.de Wilde	NT	UMK	h(p)	**OM**	
Penaeaceae					
*Olinia chimanimani* T.Shah and I.Darbysh.	EN	CHIM	s, t	**OM**	
Peraceae					
*Clutia monticola* S.Moore var. *stelleroides* (S.Moore) Radcl.‐Sm.		TSE	h(p)	**OM**	
*Clutia punctata* Wild	LC	CHIM	s	**OM**	xx
*Clutia sessilifolia* Radcl.‐Sm.	LC	CHIM	s	**OM**	xx
Phyllanthaceae					
*Moeroris manicaensis* (Radcl.‐Sm.) R.W.Bouman	VU	TSE	h(p)	**FOR**	x
*Moeroris tsetserrae* (J.F.Brunel) R.W.Bouman	CR	TSE	h(p)	**OM**	
*Phyllanthus bernierianus* Müll.Arg. var. *glaber* Radcl.‐Sm.		CHIM	s	**OM**	
Polygalaceae					
*Polygala zambesiaca* Paiva	VU	MH	s	**OM**	xx
Primulaceae					
*Lysimachia gracilipes* (P.Taylor) U.Manns and Anderb.		MH	h(p)	**OM**	
Proteaceae					
*Faurea rubriflora* Marner		MH	t	**FOR**	x
*Protea asymmetrica* Beard	NT	NYA	s	**OM**	xx
*Protea caffra* Meisn. subsp. *gazensis* (Beard) Chisumpa and Brummitt	[LC]	MH	s, t	**OM**	x
*Protea enervis* Wild	VU	CHIM	s	**OM**	
Rubiaceae					
*Anthospermum ammanioides* S.Moore	LC	MH	s	**OM**	x
*Anthospermum vallicola* S.Moore	LC	MH	s	**OM**	xx
*Anthospermum zimbabwense* Puff	NT	MH	s	**OM**	x
*Canthium oligocarpum* Hiern subsp. *angustifolium* Bridson		MH	t	**FOR**	x
*Coffea ligustroides* S.Moore	VU	MH‐S	s	**FOR**	
*Empogona jenniferae* Cheek	EN	CHIM	t	**FOR**	
*Oldenlandia cana* Bremek.	LC	CHIM	h(a)	**OM**	
*Otiophora inyangana* N.E.Br. subsp. *inyangana*		MH	h(p), s	**OM**	x
*Otiophora inyangana* N.E.Br. subsp. *parvifolia* (Verdc.) Puff		CHIM	h(p), s	**OM**	
*Otiophora lanceolata* Verdc.	VU	CHIM low elev	h(p), s	**WL**	
*Pavetta bridsoniae* P.I.Forst.	LC	NYA	s	**FOR**	
*Pavetta comostyla* S.Moore subsp. *comostyla*		MHE	s, t	**FOR**	
*Pavetta umtalensis* Bremek.	LC	MHE	s, t	**FOR**	
*Pentas zanzibarica* (Klotzsch) Vatke subsp. *zanzibarica* var. *haroniensis* Verdc.		CHIM low elev	h(p)	**FOR**	
*Rytigynia* sp. D of FZ		CHIM	s	**FOR**	
*Rytigynia* sp. E of FZ		MOC	s	**FOR**	
*Sericanthe chimanimaniensis* Wursten and De Block	VU	MH‐S	s, t	**OM**	x
*Tricalysia coriacea* (Benth.) Hiern subsp. *angustifolia* (J.G.Garcia) Robbr.	NT	MH	s, t	**FOR**	
Rutaceae					
*Vepris drummondii* Mendonça	VU	CHIM low elev	s	**FOR**	
Santalaceae					
*Thesium bundiense* Hilliard	DD	CHIM	h(p)	**OM**	
*Thesium chimanimaniense* Brenan	LC	CHIM	h(p)	**OM**	
*Thesium dolichomeres* Brenan	LC	CHIM	h(p)	**OM**	
*Thesium pygmaeum* Hilliard	LC	CHIM	h(p)	**OM**	
Sapotaceae					
*Synsepalum chimanimani* S.Rokni and I.Darbysh.	EN	CHIM low elev	s, t	**FOR**	
Scrophulariaceae					
*Hebenstretia oatesii* Rolfe subsp. *inyangana* Roesler	LC	NYA	h(p)	**OM**	x
*Jamesbrittenia carvalhoi* (Engl.) Hilliard	LC	MH	h(p), s	**OM**	x
*Selago anatrichota* Hilliard	LC	CHIM	h(p)	**OM**	
*Selago goetzei* Rolfe subsp. *ambigua* Hilliard		MH	h(p)	**OM**	x
*Selago swynnertonii* (S.Moore) Hilliard [both vars]		MH	h(p)	**OM**	x
*Selago thyrsoidea* Baker var. *austrorhodesica* Brenan	NT	NYA	h(p)	**OM**	x
Thymelaeaceae					
*Struthiola montana* B.Peterson	DD	CHIM	s	**OM**	x

*Note:* IUCN Red Listings (where available): DD = Data Deficient, LC = Least Concern, NT = Near‐Threatened, VU = Vulnerable, EN = Endangered, CR = Critically Endangered. MGU = Montane Geomorphological Unit as in Table 1. Lifeforms: cl = woody climber, h(cl) = herbaceous climber, h(epi) = herbaceous epiphyte, h(geo) = herbaceous geophyte, h(gr) = herbaceous graminoid, h(p) = herbaceous perennial suffrutex, h(succ) = herbaceous succulent, s = woody shrub, t = tree. Habitat: FOR = evergreen forest, OM = open montane, WL = savanna woodland (also see Table 4). > 2,000 m = whether taxon extends above 2,000 m elevation; entries marked with xx indicate taxa only or predominately above 2000 m. An expanded version with additional geographic detail and variables is given as Appendix 1.

**FIGURE 3:**
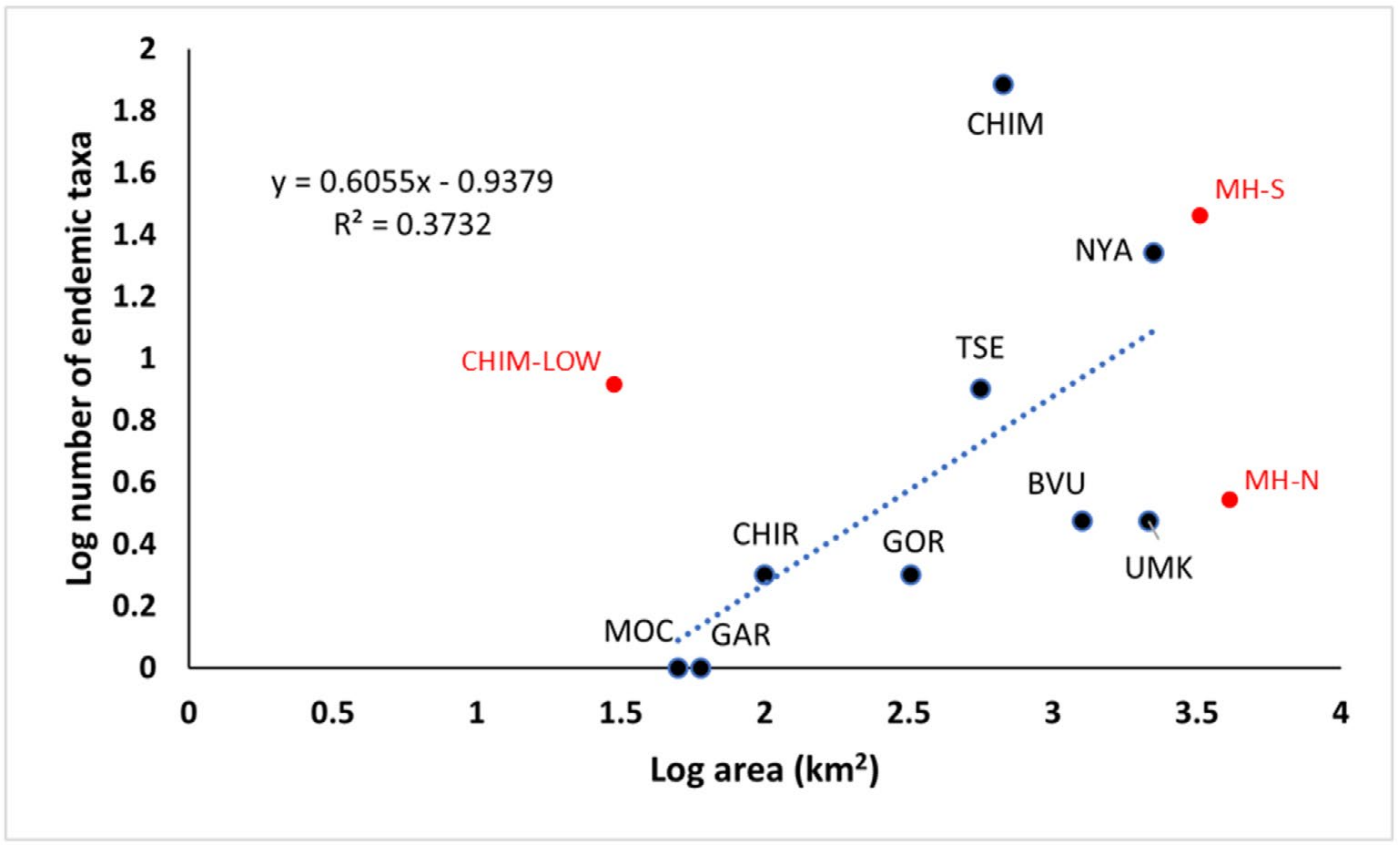
Log regression of number of endemic taxa against area for each of the Manica Highland montane geographic units (MGU). Abbreviations follow Table 3. Points in red (CHIM‐LOW, MH‐S, MH‐N) are not independent of other values so were not used in the regression calculation.

The geographic spread of endemics across the MGUs is very unequal as shown in Table 3, and a log regression (Figure 3) shows that area is a weak indicator (*R* ^2^ = 0.37) of endemic richness in each unit. The most significant sub‐centre is the Chimanimani Mountains with 77 unique endemics, 36% of the total. Nearly all of these are confined to quartzite‐derived soils with eight found only at low elevations (CHIM‐LOW in Table 3). The other significant sub‐centre is Nyanga–Rukotso–Serra Choa in the north with 22 (10.2%) strict endemics. The two central blocks (Stapleford–Mutare–Bvumba and Banti–Tsetserra–Rotanda) only have 11 endemics between them. Overall, 119 endemics (55.1%) are found only in the Southern section from Tsetserra southward, compared with 30 endemics (13.9%) confined to the slightly larger Northern section, while a further 67 (31.0%) occur across the whole area. Values for the two sections (shown in red, Figure 3) were not used in calculating the regression as they are not independent of other values.

**TABLE 3:** A summary of the numbers of plant endemics within each of the Manica Highlands montane geomorphological units (Zimbabwe–Mozambique).

Montane Geomorphological Unit (MGU)	No. of endemics	% of total
1. Nyanga–Rukotso–Serra Choa (NYA)	22	10.2
2. Mount Gorongosa (GOR)	2	0.9
3. Penhalonga–Stapleford–Bvumba (BVU)	3	1.4
4. Mount Garuso (GAR)	0	0
5. Tsetserra–Himalaya (TSE)	8	3.7
6. Eastern outliers – Serra Mocuta & Mount Madzimo (MOC)	1	0.5
7. Chimanimani Mountains (including foothills) (CHIM)	77	35.6
*[only Chimanimani foothills/lowlands on scarp side CHIM‐LOW]*	*[8]*	*[3.7]*
8. Chimanimani Uplands (Umkondo Sandstones) (UMK)	3	1.4
9. Mt. Selinda–Chirinda (CHIR)	2	0.9
10. Manica Highlands – non‐specific but only N section (MH‐N)	3	1.4
11. Manica Highlands – non‐specific but only S section (MH‐S)	28	13.0
12. Across whole range of Manica Highlands – not localised (MH)	67	31.0
Total	216	100

### 3.2 Habitat

The habitats from which each taxon has been recorded are shown in Table A1 in Appendix 1, with the amalgamated group—Open Montane (bare rock, montane grassland, scrub, and wetland), Moist Forest (evergreen and semi‐deciduous moist forest, forest margins, and riverbanks), and Woodland shown in Table 2. A very high proportion, 173 endemics or 80.1%, are found in open montane habitats, while only 36 (16.7%) are found in moist forest habitats (Table 4). More specifically, 127 (58.8%) endemic taxa can be found in montane grassland and 89 (41.2%) taxa are found on rocky outcrops and crags. Only 17 taxa are recorded from woodland, although this is possibly the most geographically widespread habitat.

**TABLE 4:** Habitat preferences of plant taxa endemic to the Manica Highlands (Zimbabwe–Mozambique).

Habitat	No. of endemics	% of total
Rock + crags [ROCK]	89	41.2
Montane grassland [MG]	127	58.8
Montane scrub [MS]	22	10.2
Moist forest–montane [MMF]	26	12.0
Moist forest–lowland [LMF]	4	1.9
Forest margins [FM]	20	9.3
River margins & forest [RIV]	5	2.3
Savanna woodland (mostly *Brachystegia*) [WL]	17	7.9
Wetland/wet grassland [WET]	8	3.7
Simplification into primary habitats		
Open montane (ROCK + MG + MS + WET) (=OM in Table 2)	173	80.1
Moist forest (MMF + LMF + FM + RIV) (=FOR in Table 2)	36	16.7
Savanna woodland (WL) (=WL in Table 2)	7	3.2
Total	216	100.0

*Note:* Numbers in first section of table do not add up to total number of endemics or 100% as many taxa occur in two or more habitat categories.

From Google Earth, the extent of open montane habitats (OM) is around 1600 km^2^ while the extent of moist forest habitats (MF) is 1770 km^2^. Of the remaining 4030 km^2^, about half is miombo woodland or secondary grassland and scrub (WL). This suggests there are 10.81 endemic taxa/100 km^2^ confined to open habitats and only 2.03 taxa/100 km^2^ in moist forest habitats, with just 0.17 endemics/100 km^2^ in woodland or transformed habitats. There were too few data points for a meaningful regression.

The majority of endemics (148, 68.5%) are herbaceous, that is, not particularly woody, and nearly all are perennial; only three are true annuals (Table A1 in Appendix 1). Of the perennial herbs, 35 (16%) are geophytes, 19 (9%) are succulents, and four are epiphytes. There are 83 taxa (38.4%) that can be woody (a few taxa can occur as shrubs or perennial herbs), although only 18 of these have the potential to become full‐size trees, and three are epiphytic parasites.

### 3.3 Elevation

Most endemic taxa have nearly all their populations or localities at elevations between 1200 and 2000 m, but for 10, primarily those associated with Chirinda Forest and the Chimanimani Uplands, the main distribution appears to be lower at 1000–1200 m (see Table A1 in Appendix 1). In addition, there are nine primarily confined to low‐lying quartzite slopes of the Chimanimani Mountains below 800 m. In contrast, 24 taxa (11.1%) are almost entirely confined to elevations over 2000 m with no records below 1700 m (Table 2, Table A1 in Appendix 1), a group of particular significance given concerns over climate change and potential elevational shifts in distribution.

### 3.4 Conservation Status

**FIGURE 4:**
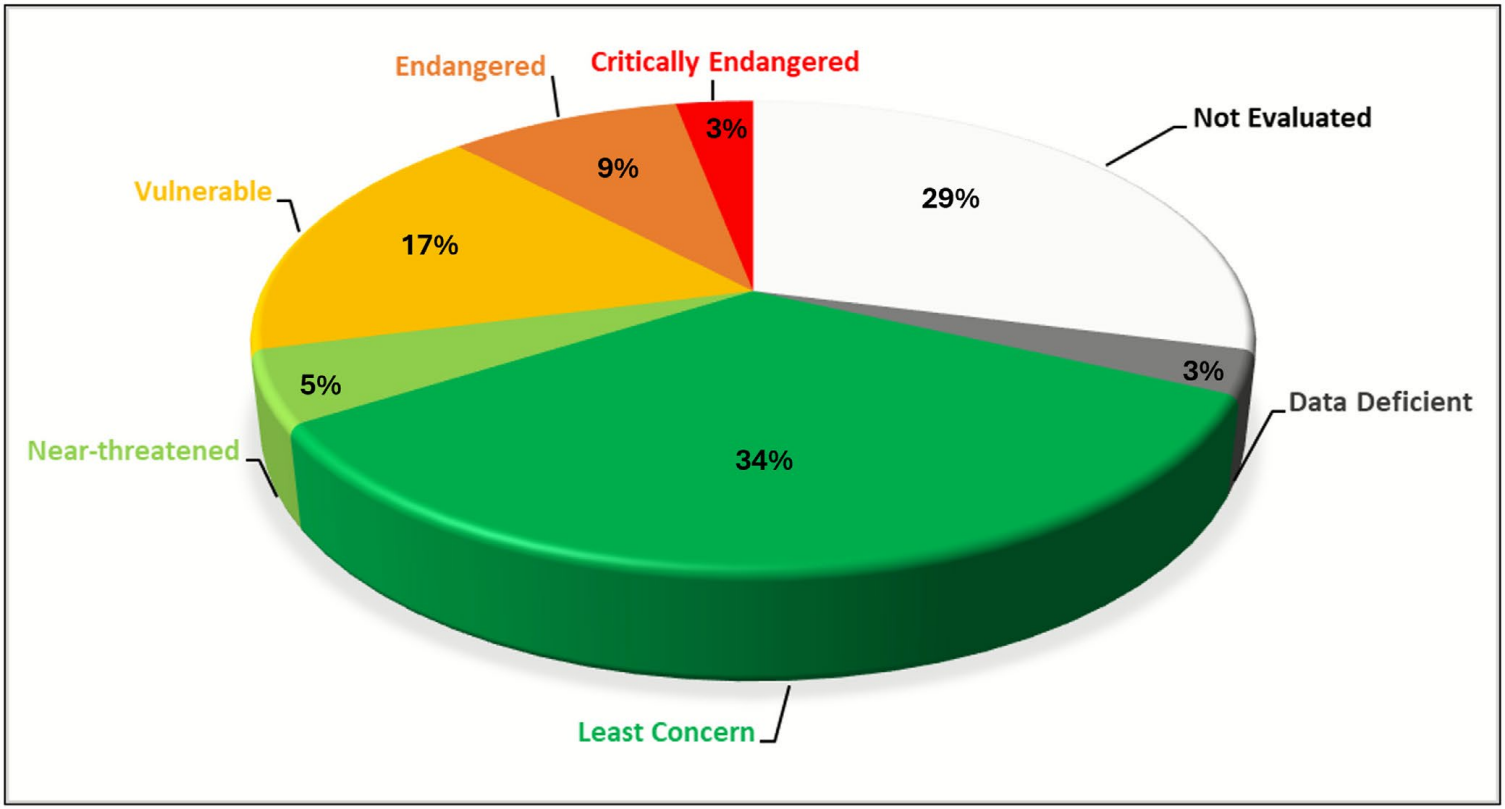
Proportions of threat categories for endemic plant taxa from the Manica Highlands (following IUCN Red List 2024).

To date only 154 (71.3%) of endemic taxa have been assessed for the IUCN Red List (IUCN 2024) with 62 still not assessed. The larger portion of those assessed are Least Concern (74 taxa) or Data Deficient (6), while almost a third (63 taxa or 29.2%) are considered threatened at some level (Critically Endangered, Endangered or Vulnerable) and 11 are Near‐Threatened (Figure 4, Table A1 in Appendix 1). However, it should be noted that some published assessments were done using earlier IUCN categories and may need to be revised.

From the threat details outlined in IUCN assessments (IUCN 2024), the main threat by far is habitat loss through afforestation and encroachment of alien invasive woody plants into montane grassland. Loss of moist forest through increased frequency of wildfire is a lesser threat, while direct impacts owing to small‐scale gold panning were only significant in one or two instances.

### 3.5 Near‐Endemics

Of the 13 identified near‐endemic taxa (Table A2 in Appendix 1), most have significant populations in uplands or hills away from the Manica Highlands, including three north of the Zambezi River (Mounts Mabu, Morrumbala, Namuli). One is an uncertain species (*Sericanthe* sp. A of *Flora Zambesiaca*, Rubiaceae) while another (*Polystachya valentina* la Croix & P.J. Cribb, Orchidaceae) is possibly also known from Mount Namuli but would otherwise be a Manica Highlands endemic. Two very significant montane shrubs, *Protea dracomontana* and *Leucospermum saxosum*, are also known from the mountains south of the Limpopo River (Maloti–Drakensberg and LMEE, respectively). In the past, the Nyanga population of *P. dracomontana* was regarded as a distinct taxon, *P. inyanganiensis* Beard (Beard 1992).

The 13 near‐endemics follow a similar ecological pattern to the endemics, being primarily (54%) herbaceous taxa found at mid‐elevations in open montane habitats, while five are trees or shrubs. Only one near‐endemic (*P. dracomontana*) is found primarily above 2000 m elevation on Mount Nyangani (Beard 1992). Ten taxa have a published IUCN threat status, most being Least Concern with just three either Endangered or Vulnerable.

## 4 Discussion

### 4.1 The Manica Highlands Centre of Floristic Endemism

Based on the number of plant endemic taxa recorded and their distribution patterns across the mountains, we replace the Chimanimani–Nyanga Centre of van Wyk and Smith (2001) with a smaller but better‐defined Manica Highlands Centre. The main differences between the two studies arose because of the limited data available at the time on which taxa were endemic and their full distributions. For example, some of their Chimanimani–Nyanga Centre in fact comprises mid‐altitude plateaux or lowland gaps and are now excluded, and we here include Chirinda Forest and some adjacent massifs such as Mount Gorongosa, the latter owing to large numbers of endemics shared with the main block. However, we confirm their internal Chimanimani and Nyanga secondary concentrations of endemics as sub‐centres. The Manica Highlands Centre thus has a revised, longer north–south extent of 300 km and an area of 7400 km^2^. Importantly, we more than double van Wyk & Smith's estimate of endemics from 100 to 216 taxa.

Geographically, the revised Centre lies between Mucina and Rutherford's (2006) Northern Sourveld Endemics unit south of the Limpopo River (Clark et al. 2011a) and centres of endemism to the north such as the Mulanje–Namuli Centre, which has been incorporated into the South East Africa Montane Archipelago ecoregion (Bayliss et al. 2024). The Northern Sourveld Endemics area is part of a continuum of nutrient‐poor, grassland‐dominated, mountain‐centred endemism found along the eastern Great Escarpment in southern Africa where grassland and associated open habitats are spatially dominant over forest and woodland (Clark and Barker 2014; Clark et al. 2014, 2022), while the Mulanje–Namuli Centre covers a series of 30 discrete granitic inselbergs or massifs with similar geomorphology and vegetation types totaling around 2100 km^2^ above 1000 m elevation across northern Mozambique and southern Malawi (Bayliss et al. 2024). The latter Centre also includes significant areas of open montane habitat, including grassland (especially on Mounts Mulanje and Namuli) and bare rock, similar to that seen in the Manica Highlands. Our revised Centre contains many temperate botanical elements and forms a southern limit for plant endemics associated with a wider tropical high‐elevation guild (Wild 1956).

At a broader scale, and based primarily on woody species composition, White (1978, 1981) refers to an island‐like Afromontane archipelago as being distinct from the surrounding lowland vegetation types. Many woody Afromontane endemics are found across large parts of montane Africa and show little local endemism (White 1983). However, it appears that montane grassland, shrubland, and crag species are much less widely distributed, with more local endemism. White (1978) did make especial mention of the Chimanimani Mountains “island” based on lists in Goodier and Phipps (1961) and Wild (1964), perhaps recognizing its floral uniqueness.

### 4.2 Levels of Endemism

With an estimated total regional flora of 1800–2300 taxa (authors' estimates) and endemism at around nine to 12%, we confirm that in terms of number of endemic taxa the Manica Highlands are the third richest mountain system in summer‐rainfall southern Africa (i.e., outside of the winter‐rainfall Cape region) (Table 5), comparable to the much larger Drakensberg Mountain Centre (9% endemism, Carbutt 2019) and possibly second only to the six‐times larger LMEE (10.7% endemism, Clark et al. 2022). Our endemism figure is significantly higher than the 6.7% endemism originally estimated for the Chimanimani–Nyanga Centre by van Wyk and Smith (2001). With 2.92 endemics/100 km^2^ the Manica Highlands have the third highest density of endemics (Table 5) after the much smaller Mount Mulanje in Malawi and the Mulanje–Namuli inselberg area (which incorporates Mount Mulanje) at 6.05 endemics/100 km^2^, and significantly richer than the larger LMEE at 0.91/endemics/100km^2^ and the Drakensberg Mountain Centre at 0.64 endemics/100 km^2^.

**TABLE 5:** Endemism and plant diversity in various southern African mountain areas, from north to south.

Massif/Mountain area	Total flora	Total endemics	% endemism	Surface area (km^2^)	Endemics/100 km^2^	References
Nyika Plateau (Malawi–Zambia)–NYI	1891	33	1.7	1800	1.83	Burrows and Willis (2005)
South East Africa Montane Archipelago Ecoregion (Malawi–Mozambique)–SEAMA	1500–1800	127	7.1–8.5	2100	6.05	Bayliss et al. (2024)
Mount Mulanje (Malawi) (a component of SEAMA)–MUL	1319	48	3.6	640	7.50	Bayliss et al. (2024), revision of Strugnell (2006)
Manica Highlands Centre of Floristic Endemism (Zimbabwe–Mozambique)–MH	1800–2300	216	9.4–12.0	7400	2.92	This paper
Soutpansberg Centre of Floristic Endemism (South Africa)–SOU	2454	44	1.8	6700	0.65	Hahn (2017)
Limpopo–Mpumalanga–Eswatini Escarpment (South Africa–Eswatini)–LMEE	4657	496	10.7	53,954	0.91	Clark et al. (2022)
Wolkberg Centre of Floristic Endemism (South Africa) (a component in LMEE)–WOL	c.2500	130	5.2	5980	2.17	Cowling and Hilton Taylor (1994), van Wyk and Smith (2001)
Drakensberg Mountain Centre of Floristic Endemism (South Africa–Lesotho)–DMC	2520	235	9.0	36,478	0.64	Carbutt (2019)
Great Winterberg–Amatholes (South Africa)–GWA	1878	35	1.9	7382	0.47	Clark et al. (2014)

*Note:* The Wolkberg centre of floristic endemism is a subset of the larger Limpopo–Mpumalanga–Eswatini Escarpment.

**FIGURE 5:**
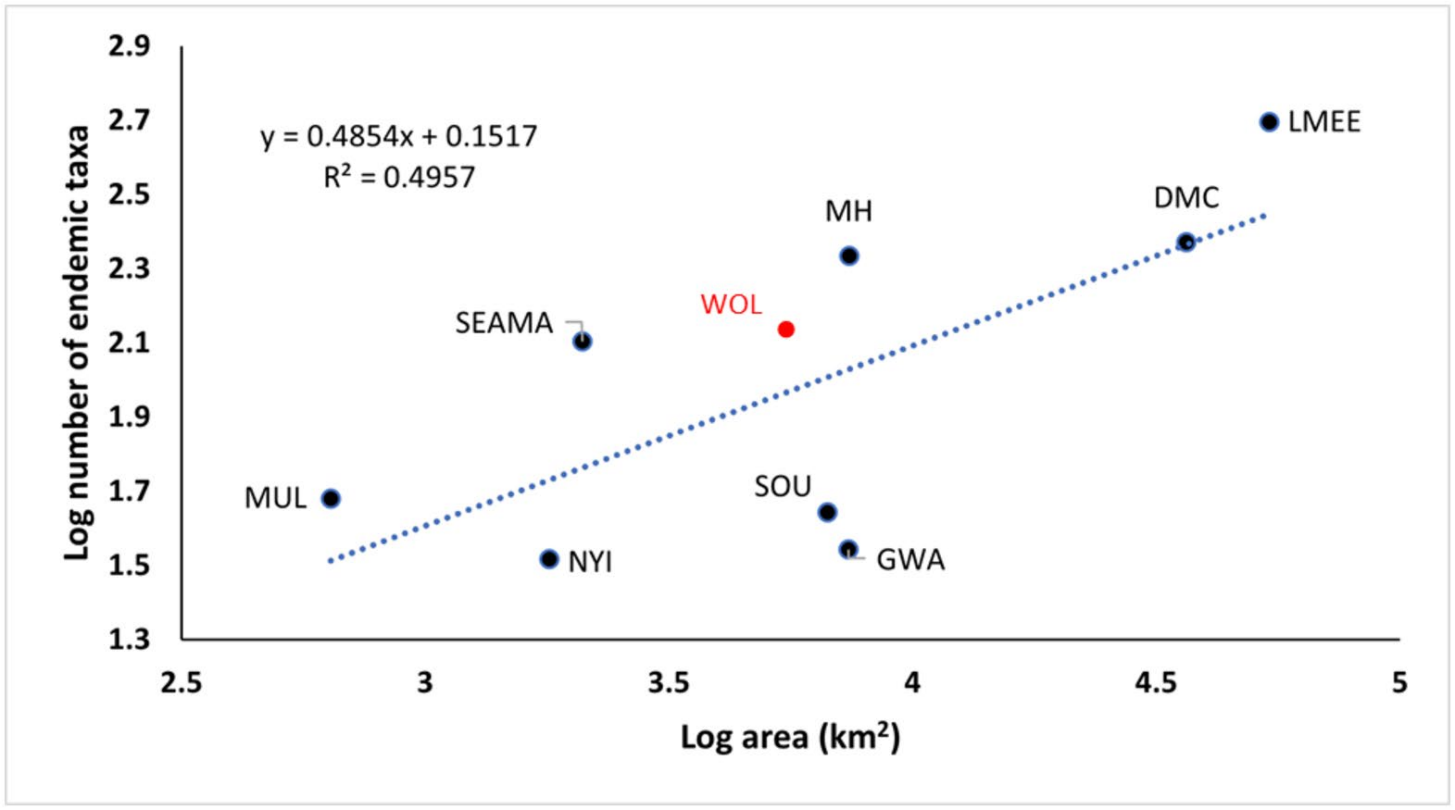
Log regression of number of endemic taxa for nine southern African centres of floristic endemism against area extent. Abbreviations follow Table 5. Point in red (WOL) was not used in the regression calculation as it is a subset of LMEE.

When assessed in terms of extent and using a simple log regression (Figure 5), area is seen to be only a partial predictor (*R* ^2^ = 5) of endemic richness in southern African mountains, although slightly stronger in predictive value than for MGUs within the Manica Highlands. This indicates that there are other important drivers of endemism in summer rainfall southern African mountains at a regional level.

In part, this high level of endemism is a result of particular or unique geology and edaphic (soil) conditions. Although most of the Manica Highlands is underlain by syenite and granite, which generally create landscapes of rounded batholiths rather than craggy outcrops, over 35% of the Manica Highland endemics are confined to the Chimanimani Mountains. Nearly all of these occur on particularly nutrient‐poor substrates derived from Umkondo quartzite (Phipps and Goodier 1962; Wild 1964; Timberlake, Darbyshire, Wursten, et al. 2016), with just two endemics (*Syncolostemon flabelliformis* (S.Moore) A.J.Paton and *Morella chimanimaniana* Verdc. & Polhill) being confined to schist substrates (Timberlake, Darbyshire, Wursten, et al. 2016).

Parallels with mountains south of the Limpopo River can be seen in the LMEE with its strong edaphic partitioning of endemics on Barberton serpentines, Black Reef quartzites (with the only other population of *Leucospermum saxosum* outside of the Chimanimanis; van Wyk and Smith 2001), Sekhukhune ultramafics and Chuniespoort dolomites (van Wyk and Smith 2001; Mucina and Rutherford 2006; Clark et al. 2022), and Karoo Supergroup sedimentary and volcanic intrusive substrates in the Hantam–Roggeveld area (Clark et al. 2011b). Both the LMEE and Hantam–Roggeveld have surprisingly high endemic floras given their relatively simple topographic heterogeneity and low absolute elevation (1800–2200 m) compared to mountains of similar extent such as the Maloti–Drakensberg which are much higher (> 3000 m) and more rugged and where edaphic partitioning is not particularly evident (Carbutt 2019).

### 4.3 Particular Areas of Endemism

As mentioned above, the Chimanimani Mountains are confirmed as being by far the most significant focal point for endemism within the Centre (Table 3). Covering just a small proportion (9%) of the Centre, the Chimanimanis support 139 (64%) of Manica Highland endemics (see Table A1 in Appendix 1) with a very high density of 20.7 endemics/100 km^2^, 77 of these being strict local endemics. As mentioned above, this probably results from the rugged quartzite topography and associated habitat diversity.

The second‐richest unit for endemism is the Nyanga area with 86 endemics (Table A1 in Appendix 1) or 3.9 endemics/100 km^2^, of which 22 are strict local endemics (Table 3). This area has a large extent of montane grassland compared with other MGUs, apart from Chimanimani. As discussed below, it is these open habitats, especially grassland and bare rock, that appear to be so important in causing or maintaining local endemism.

We recognize that sampling effort is not similar across the various MGUs, with some areas being better known (e.g., Nyanga, Bvumba, Chimanimani Mountains and Chirinda Forest, see Clark et al. 2017, Timberlake et al. 2020, Timberlake, Darbyshire, Wursten, et al. 2016 and Timberlake and Shaw 1994, respectively), while other areas such as Serra Choa, Mount Garuso, Tsetserra, and Serra Mocuta have been very under‐collected. Further collecting effort in these areas (e.g., see Osborne and Matimele 2018) may well raise their importance as regards endemics, especially those with unusual substrates. However, the overwhelming importance of the Chimanimani Mountains as a sub‐centre of endemism is unlikely to diminish.

### 4.4 Habitats

A major finding of this study concerns the habitat preferences of endemics. The habitats of greatest importance are montane grassland and bare rock + rocky crags (ROCK) (Table 4, Table A1 in Appendix 1) rather than more closed habitats such as forest and forest margins (FOR). Montane grassland is of especial significance, particularly in the Chimanimani and Nyanga areas, with more than half the endemics being recorded from there. Overall, endemics found in montane grassland, montane scrub, and on bare rock form the core of the Centre and best typify it.

However, as many species have more than one habitat indicated across their records, when specific habitats were grouped into open montane habitats (OM) and forest habitats (FOR; see Table 4), it can be seen that of the 216 endemic taxa, 80.1% are primarily found in open montane habitats, although the approximate extent of these habitats is around 1600 km^2^ or 22%. This is in contrast to around 17% of endemics being found in forest and densely wooded habitats, yet the extent is similar at 1770 km^2^ or 24%. Miombo and similar woodlands are widespread both within the Centre and in many surrounding areas, but only 3.2% of endemic taxa are found here. Possible reasons for this interesting finding are discussed further below. Although a simple log regression of these data was done, the lack of separate data points makes it unreliable.

It could be suggested that some habitats across the Centre have been much better collected or surveyed than others. Although no data exist, in proportional terms this is not thought to be significant. Woodland, the broad habitat with the least number of endemics, is generally more accessible and has probably been better collected across the whole Manicaland area than other habitats. The Nyanga, Chimanimani, and Mount Gorongosa grasslands have been of interest to botanists for many years and are moderately well collected, but probably no more so than other open montane habitats here. On the other hand, moist forests have been well studied, particularly in Zimbabwe (Müller 1999, 2006, but see Timberlake, Darbyshire, Cheek, et al. 2016 for Mozambique), yet still show lower levels of endemism than montane grasslands and rocky areas (Table 4). We do not think that the high number of endemics in open habitats is a result of differential collecting effort or differences in accessibility.

The dominance of endemics from open habitats, coupled with perennial herbs being the dominant lifeform, emphasizes the important evolutionary role these non‐wooded montane habitats have probably played in local endemism in southern Africa. This contrasts with the general perception that closed habitats such as moist forest should be priorities for conservation and restoration efforts, despite the risk to conservation of underplaying the importance of grasslands (Bond 2016; Bond et al. 2019; Silveira et al. 2020; Stevens and Bond 2024). Our assessment shows that open habitat endemics are the most numerous, provide the greatest endemic richness, and are most at risk from afforestation for timber or carbon‐credit schemes or alien invasive plants, threats which mostly occur in grasslands. This complements similar results from other southern African mountains for both fauna and flora (e.g., Armstrong and van Hensbergen 1999; Clark et al. 2011a, 2022; Bond 2016).

### 4.5 Altitude and Climate Change

The highest elevation zone of the Manica Highlands (> 2000 m) is considered part of the ancient Gondwana erosional land surface dating from the late Jurassic around 150 mya, while the rest at lower elevations (> 1200 m) forms part of the post‐Gondwana or African erosion surfaces dating from 140 to 26 mya (Lister 1987). Together these form part of the Gondwana and Africa paleo‐land surfaces of Partridge and Maud (1987) and Moore et al. (2017), and it is at these elevations that most endemics are found.

At around 250 km^2^ (unpublished data), the extent of the Centre above an elevation of 2000 m is quite small (3.5% of total extent), yet supports a disproportionate number (102 or 47.2%) of the 216 endemic taxa; 24 of these being primarily confined to this zone (Table 2). In the Nyanga area in particular, 10 out of 22 endemics are found at elevations above 2000 m, especially on the dolerite sills of Mount Nyangani, World's View–Troutbeck and Rukotso; only four of the Nyanga endemics are primarily recorded from below 2000 m. This finding is of conservation significance in light of potential altitudinal shifts in range resulting from climate change. On the other hand, from threat descriptors in the IUCN Red List assessments, taxa occurring at these high elevations are less threatened by wildfires, land transformation and alien invasive species.

Climate change is an as yet unquantified risk to these endemics. Interestingly, an early analysis of changes in distribution of montane vegetation in Zimbabwe under scenarios of 50% and 150% of current rainfall (Wild 1968) suggested virtually no change in its extent. But such stability is unlikely to be the case in practice, especially as minimum temperatures will also change under current climate change scenarios (e.g., CSIR 2019). The installation of weather stations in the Nyanga and Bvumba areas by the African Mountain Research Foundation in 2022 (e.g., https://jollyweather.com/nyanga/index.html) now provides an opportunity to determine actual changes using meteorological variables over time.

### 4.6 Causes of Endemism

A major question is what might be the causes of such high levels of endemism. For the Manica Highlands Centre this is not immediately obvious as both the area and the separate MGUs within it are not particularly geographically separated from the surrounding landscape (e.g., the Mashonaland plateau in Zimbabwe to the west) and neither are the topographic differences very marked. In addition, with the clear exception of the Chimanimani Mountains (Watson 1969), the underlying geology is similar across the Centre and on the surrounding Mashonaland and Manicaland plateaux (Watson 1969; Stagman 1978; ING 1987). Endemism to the Chimanimani sub‐centre, however, would seem to be almost entirely based on the nutrient‐poor quartzite substrate (Phipps and Goodier 1962; Wild 1964; Timberlake, Darbyshire, Wursten, et al. 2016), a form of ecological isolation. Out of the 77 listed Chimanimani Mountain endemics, only two are confined to schist substrates (Timberlake, Darbyshire, Wursten, et al. 2016) with the rest confined to quartzite.

The evolutionary ages of these local endemics are not yet known as genomic data for these taxa and many near‐relatives are not available, although this could form the basis of a future, comprehensive study. We assume that the majority are neo‐endemics, that is, they have evolved over the last few million years from more‐widely distributed ancestors. For example, Wild (1964) suggests that many of the Chimanimani endemics are what he terms vicarious, with their nearest relatives having a wider distribution, while Fjeldså and Lovett (1997) suggest that many inselberg endemics from the Eastern Arc mountains in Tanzania are neo‐endemics.

It has often been stated in southern Africa that the original vegetation was Afromontane forest and that montane grasslands and scrub have arisen mostly due to human clearance and fire (e.g., White 1978), perhaps with a Late Pleistocene origin. But this does not explain the large number of endemics found in these habitats, which must have been in place for millennia and long pre‐date any human impacts. It is possible that a significant extent of grassland, scrub and rocky or crag habitat has been present for some millions of years (possibly since the Miocene, 20 mya), a conclusion also arrived at by Meadows and Linder (1993) for grasslands on the Nyika plateau in northern Malawi. However, it is also possible that, as a result of more frequent human‐made fires over the last thousand years or so, the extent of open habitats and thus the populations of many endemic species have now increased and their status improved.

These findings support the theory on old, climatically buffered, infertile landscapes (OCBIL) developed for Western Australia (Hopper 2009; Hopper et al. 2021). OCBIL landscapes are geologically ancient with infertile soils (especially for phosphorus) and lie in more climatically stable areas within 500 km of the coast (Hopper 2009, Hopper et al. 2021), in contrast to young, often disturbed, fertile landscapes (YODFEL) with significantly higher plant productivity which are more typical of the surrounding valleys and lowlands. Plant species diversity is said to be significantly higher in such impoverished landscapes with more species of restricted distribution (Hopper et al. 2021), thought to be due partly to reduced dispersal possibilities resulting from the physiological costs—growth rates are slower owing to the low nutrient status. Another factor increasing species diversity is said to be the persistence of taxa due to relative environmental stability and low disturbance regimes (Hopper et al. 2021). Together, these factors help explain the diversity and endemism levels of the flora of the Western Cape in South Africa (Hopper et al. 2021) and, at a much smaller scale, that associated with the Centre.

Therefore, we suggest that it is nutrient stress through the low availability of soil nutrients, forcing adaptation for plants to survive, which is the probable cause of much of the speciation and endemism found in the Manica Highlands in addition to the topographic diversity created by erosion‐resistant rocks and, at the highest elevations, the climatic stressors of low temperatures and large diurnal fluctuations.

Future‐intensive fieldwork on the area's endemic species is required to clarify exactly what the drivers of endemism might be, including the substrates on which they occur and phylogenetic analyses of the species and their nearest relatives.

### 4.7 Conservation and Threats

Overall, the Centre is comparatively well‐protected with 2312 km^2^ under formal State protection, about a third of its total area. Within Zimbabwe much is designated as National Park, Forest Land or Botanical Reserve while a significant part is under enlightened private conservation management, such as in the Troutbeck–Kwaraguza area of Nyanga and the Bvumba. Protection in the Mozambique portion is far more limited, with just the Chimanimani Mountains gazetted as a National Park while Mount Gorongosa forms part of Gorongosa National Park. However, in the 1950s and 1960s, large areas of grassland and scrub at medium altitude in Zimbabwe were cleared for pine and wattle plantations (see below), especially south of Nyanga and in the Chimanimani and Chipinge districts. Much endemic‐rich grassland habitat appears to have been lost at that time. Nearly all the indigenous moist forest plots in Zimbabwe recorded in the 1970s and covering the full range of forest types (Müller 1999, 2006; Timberlake et al. 2021) lie within the Centre and these appear to be still relatively intact 50 years later (Timberlake and Müller 2021). Compared to the Mulanje–Namuli Centre (Bayliss et al. 2024), where there is far less formal protection and a much greater rate of habitat clearance and impact of fire, the conservation status of nearly all Manica Highlands endemics appears to be much better.

The main threats to montane biodiversity within the Manica Highlands at present lie in the invasion of alien invasive plants into montane grassland (van Wyk and Smith 2001; Clark et al. 2017, 2019) and, to a lesser extent, the assumed increase in the frequency of wildfires (Timberlake, Darbyshire, Wursten, et al. 2016) leading to repeated loss of regenerants and suppression of the woody flora. The principal alien invasives are woody species including *Pinus* (especially *P. patula* Schltdl. & Cham., Pinaceae) and Australian wattles ( *Acacia dealbata* Link, *A. mearnsii* De Wild., Fabaceae) in the montane grasslands, especially in Nyanga, although other species such as *Hedychium gardnerianum* Ker Gawl. (Zingiberaceae), *Psidium guajava* L. (Myrtaceae), and *Lantana camara* L. (Verbenaceae) are widespread in the Bvumba (Timberlake et al. 2020). The shrub *Vernonanthura polyanthes* (Spreng.) Vega & M.Dematteis (Asteraceae) has spread rapidly in cleared forest areas in recent years (Timberlake, Darbyshire, Cheek, et al. 2016; Timberlake, Darbyshire, Wursten, et al. 2016; Timberlake et al. 2020) and poses a major invasive threat at medium and lower altitudes. Secondary concerns are illegal, artisanal gold mining, especially in the Chimanimani Mountains and at lower altitudes in Mozambique (Timberlake, Darbyshire, Wursten, et al. 2016), although this is not considered to be a major direct threat as it tends to be localized and stream‐sides are not a major habitat for endemics.

It is not yet clear what effect these threats will eventually have on the endemic flora of the Manica Highlands but, for example, Childes (1997) indicated that 40% of Nyanga National Park was affected by invasives in 1988 and the situation appears to have worsened since then (S. Childes, pers. comm.). Open habitats such as montane grassland and scrub, the very habitats that support most endemics, are most under threat from both alien invasives and fire.

Looking to the future, climate change is an unquantified risk and one that needs to be investigated more specifically (Nsengiyumva 2019). Species primarily found above 2000 m altitude are liable to lose much of their suitable habitat extent over the next century in the face of competition from better‐adapted species, and some may well become extinct.

## 5 Conclusion

The Manica Highlands is clearly confirmed as a very significant centre of plant endemism, one which is here termed the Manica Highlands Centre of Floristic Endemism, part of a chain of such centres associated with the Great Escarpment. At around 9% to 12%, the level of endemism as a proportion of the estimated local flora is particularly high, one of the highest associated with the Great Escarpment and exceeded only by the Limpopo–Mpumalanga–Eswatini Escarpment area. The extent and composition of the Manica Highlands Centre is here quantified and revised from that described by van Wyk and Smith (2001).

The other major finding is that the majority of endemics are found in open habitats such as montane grassland, rocky crags and montane scrub and not in closed vegetation types such as moist forest or woodland, although these habitats are equally common across the area. This is a similar finding to that from the Mulanje–Namuli centre of endemism and Limpopo–Mpumalanga–Eswatini Escarpment, as well as those sections of the eastern Great Escarpment for which there are quantified data, but runs contrary to what is often assumed in conservation planning, which is that moist forests are the most important habitat for conservation. We emphasize the great importance of montane grassland, an often‐overlooked habitat and one threatened by both alien invasive species such as *Pinus* as well as frequent fires. Future conservation priorities and funding will need to be directed at montane grasslands if countries' obligations under the Convention on Biological Diversity are to be fulfilled and the full range of montane biodiversity protected.

## Author Contributions

**Jonathan Timberlake:** conceptualization (equal), data curation (equal), formal analysis (equal), investigation (equal), methodology (equal), project administration (equal), resources (equal), writing – original draft (equal), writing – review and editing (equal). **Vincent Ralph Clark:** conceptualization (equal), formal analysis (equal), funding acquisition (lead), investigation (equal), methodology (equal), project administration (equal), resources (equal), validation (equal), writing – original draft (equal), writing – review and editing (equal).

## Funding

This work was supported by Afromontane Research Unit, University of the Free State, Sabbatical support. National Research Foundation (NRF), Scarce Skills Post‐Doctoral Fellowship (2014–2016).

## Conflicts of Interest

The authors declare no conflicts of interest.

## Data Availability

All the summarized data used in this paper are presented as Table A1 in Appendix 1, and are submitted and uploaded as part of the submission for review and publication.

## Appendix 1

Plant taxa endemic (Table A1 in Appendix 1), near‐endemic (Table A2 in Appendix 1), and excluded from consideration (Table A3 in Appendix 1), Manica Highlands (Zimbabwe–Mozambique). IUCN assessment: n/a = taxa not assessed; unpublished IUCN Red List assessments are in square brackets. Lifeform: see Table 2 in text. Habitat groups: see Table 4 in text. Above 2000 m: 1 = taxa extending over 2000 m; * = those mostly occurring above 2000 m elevation. MGU = montane geomorphological units as in Table 1 in text. Taxonomically unclear taxa in Tables A2 and A3 in Appendix 1 are highlighted in beige.

**TABLE A1:** Manica Highlands endemics.

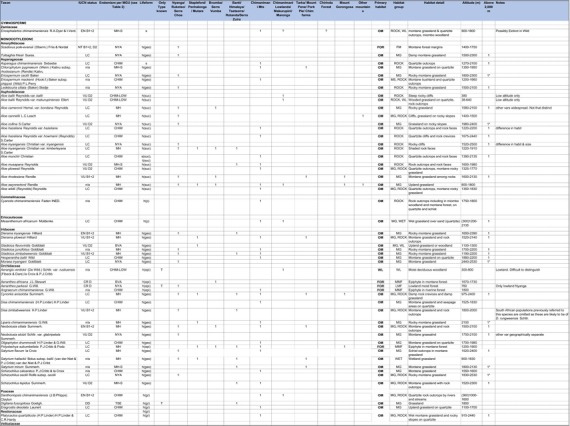

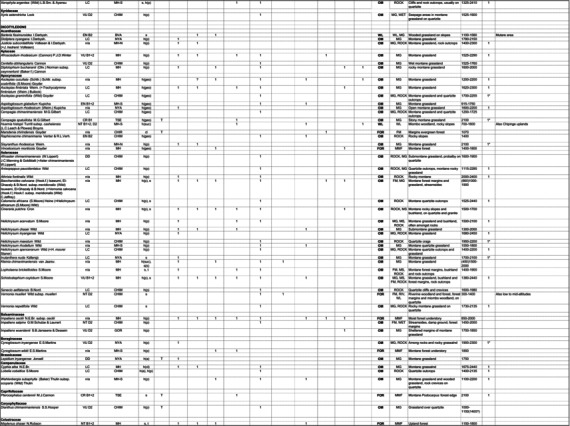

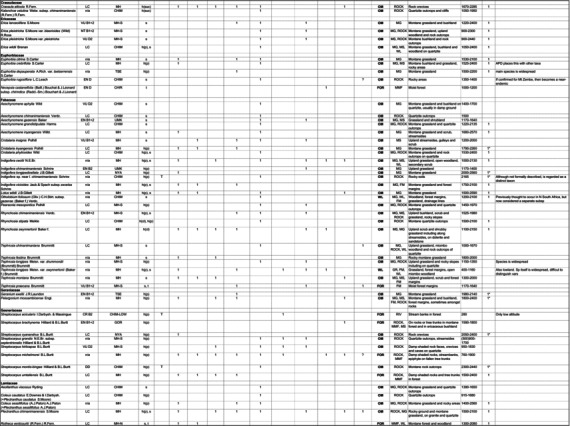

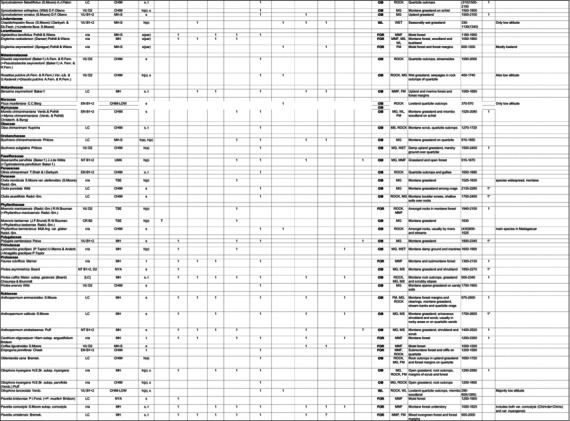

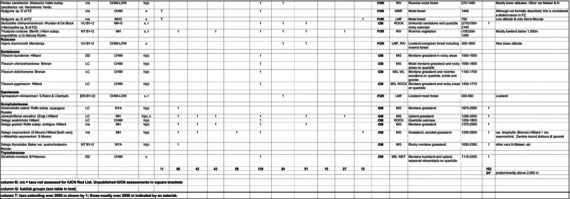

**TABLE A2:** Manica Highlands near‐endemics.

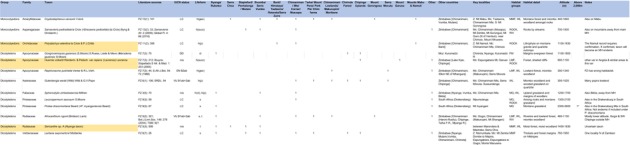

**TABLE A3:** Taxa omitted from Manica Highlands endemics list.
